# Phenylethynylbenzyl-modified biguanides inhibit pancreatic cancer tumor growth

**DOI:** 10.1038/s41598-021-87993-3

**Published:** 2021-05-10

**Authors:** Audrey Hébert, Maxime Parisotto, Marie-Camille Rowell, Alexandra Doré, Ana Fernandez Ruiz, Guillaume Lefrançois, Paloma Kalegari, Gerardo Ferbeyre, Andreea R. Schmitzer

**Affiliations:** 1 Département de Chimie-Faculté des Arts et des Sciences, Université de Montréal, 2900 Edouard Montpetit, Succursale Centre-Ville, CP 6128, Montreal, QC H3C3J7 Canada; 2 Département de Biochimie et Médecine Moléculaire, and CRCHUM-Faculté de Médecine, Université de Montréal, Montreal, QC Canada

**Keywords:** Drug development, CNS cancer

## Abstract

We present the design and synthesis of a small library of substituted biguanidium salts and their capacity to inhibit the growth of pancreatic cancer cells. We first present their in vitro and membrane activity, before we address their mechanism of action in living cells and in vivo activity. We show that phenylethynyl biguanidium salts possess higher ability to cross hydrophobic barriers, improve mitochondrial accumulation and anticancer activity. Mechanistically, the most active compound, **1b**, like metformin, activated AMPK, decreased the NAD^+^/NADH ratio and mitochondrial respiration, but at 800-fold lower concentration. In vivo studies show that compound 1b significantly inhibits the growth of pancreatic cancer xenografts in mice, while biguanides currently in clinical trials had little activity.

## Introduction

Although they have been used for decades in the treatment of type II diabetes, it is only quite recently that biguanides have been found to have interesting anticancer properties^1^. Many epidemiological studies have linked the long-term regular intake of metformin (1,1-dimethylbiguanide) to the reduction in the incidence of a variety of cancers in diabetic patients^2^. These results suggest that biguanides have anticancer activity, an idea that has been experimentally verified using cancer models in cell culture and mice^3^. However, several clinical trials featuring metformin as a chemotherapeutic agent in humans have been unsuccessful^4–6^. One of the reasons brought forward to explain this failure is the high hydrophilicity of metformin, administrated as a monoprotonated chloride salt at physiological pH (pKa 2.8 and 11.5). While animal models were given very high quantities of metformin to observe a potent anticancer activity^7^, the antidiabetic doses used in human patients were deemed too low to attain the reported antiproliferative concentration of 5 mM in vitro^8,9^.

Even if the molecular target of biguanides has not been identified yet, it is obvious that membrane insertion and mitochondrial penetration are critical for their activity^7,10^. Different metformin analogues with lipophilic substituents and improved cellular penetration were previously reported. Narise et al*.* proposed in 2014 a series of phenformin derivatives possessing various substituents on the phenyl ring and bioisosteric replacements of the biguanide unit^11^. Neuzil et al*.* showed that biguanides functionalized with a mitochondria-targeting moiety such as triphenylphosphonium (TPP^+^), possess anticancer activities up to a 800-fold higher than metformin^12^.

We have actively investigated the membrane perturbation properties of synthetic amphiphilic cationic ion transporters and antibiotics, including derivatives of phenylethynylbenzyl (PEB)—disubstituted imidazolium and benzimidazolium salts. We have demonstrated that the activities of these organic salts in artificial phospholipid bilayers of living prokaryotic and eukaryotic cells were the results of membrane penetration, self-assembly and partition^13^. The inner membrane of mitochondria, like the bacterial membrane is adapted to allow oxidative phosphorylation and electron transport. The lipid composition of mitochondria is consequently similar to bacterial membranes and includes a high phosphatidylglycerol/phosphatidylethanolamine ratio, abundant cardiolipin and very low levels of sterols^14^. We thus reasoned that conjugating biguanides with the PEB unit capable of penetrating bacterial membranes could improve metformin’s cellular/mitochondrial uptake. We generated a small library of PEB-substituted biguanidium salts and their hydrogenated analogues and studied their capacity to affect the growth of pancreatic cancer cells. We identified a novel class of biguanide compounds with better membrane crossing abilities and more potent anticancer activity than metformin and phenformin.

## Results and discussion

**Figure 1:**
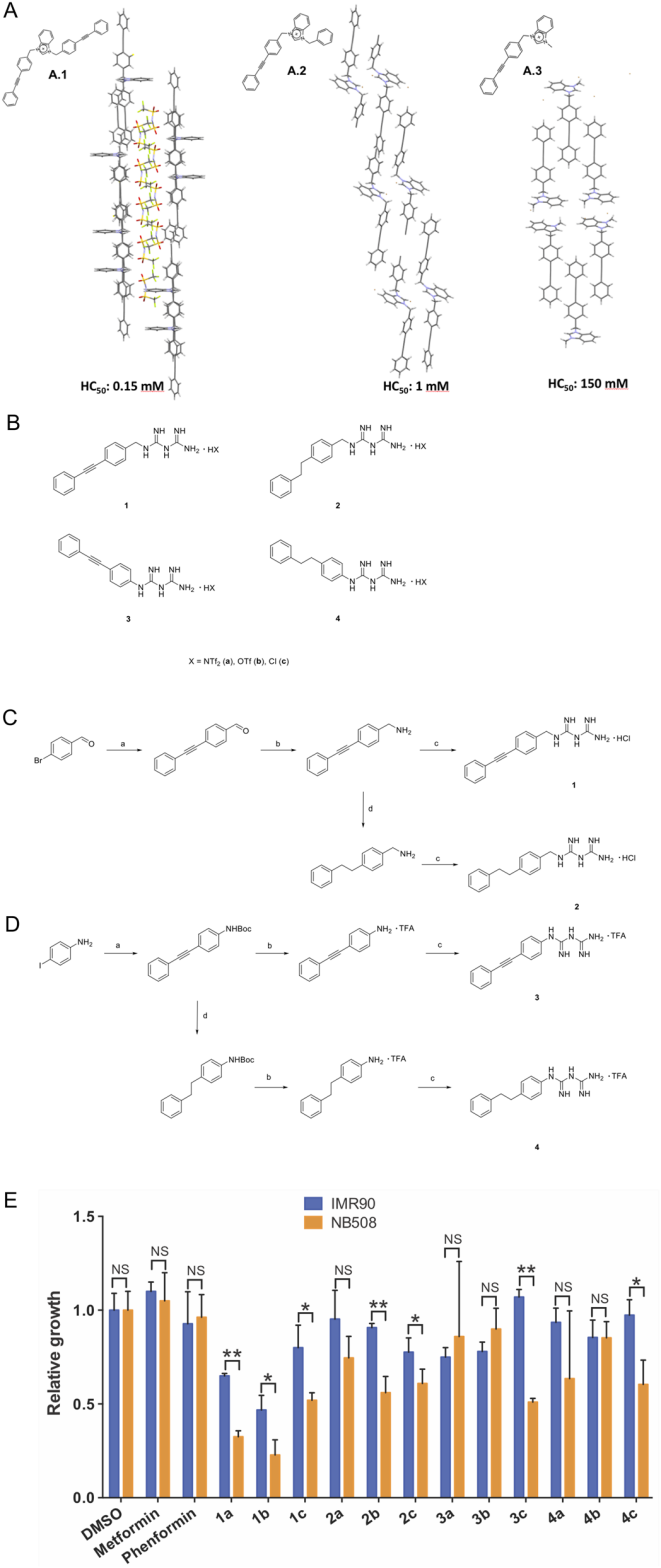
(**A**) Structure and crystal packing of previously studied PEB-disubstituted (A.1) and monosubstituted (A.2 and A.3) benzimidazolium salts^13^. Compound **A.1** is an efficient chloride transporter forming channels and generating holes in bacterial membranes and red blood cellular membranes. Compound **A.2** penetrates phospholipid membranes but is less efficient as ion transporter as it forms more compact aggregates; its toxicity on RBC is lower than compound **A.1**. The replacement of the phenyl group with a methyl group in compound **A.3** results in the formation of even more compact aggregates with even lower toxicity on RBC. (**B**) Structure of PEB-substituted biguanidium salts. For each biguanide we prepared three series of biguanidium salts with different counterions. (**C**) Synthesis of 4-(phenylethynyl)benzylbiguanide 1 and 4-(phenylethyl)benzylbiguanide 2; (a) Phenylacetylene, PdCl_2_PPh_3_, CuI, PPh_3_, Et_3_N, THF, 70 °C, o.n. (b) NaCN(BH_3_)_3_, EtOHNH_4_OAc sat./NH_3_, 80 °C, o.n (c) Dicyandiamide, TMSCl, THFanh., 145 °C, 1 h (d) Pd/C 10%, H_2_, EtOH/AcOEt, 60 °C, 2 h. (**D**) Synthesis of 4-(phenylethynylphenyl)biguanidium 3 and 4-(phenylethylphenyl)biguanidium 4 (a) (1) Boc_2_O, THF, 2 h, (2) Phenylacetylene, PdCl_2_PPh_3_, CuI, PPH_3_, Et_3_N, THF, 70°, o.n. (b) TFA, DCM, 60 °C, 2 h (c) Dicyandiamide, TMSCl, THF_anh_, 145 °C, 1 h (d) Pd/C 10%, H_2_, EtOH/AcOEt, 60 °C, 2 h. The NMR spectra of the synthesized compounds are given in Supplementary Figs. S1–S24. (**E**) Relative growth of NB508 mouse pancreatic ductal adenocarcinoma cells and IMR90 fibroblasts exposed to 5 µM of PEB-biguanidium salts. Cells were incubated for 72 h at 37 °C. Errors bars represent the standard error of the mean (SEM), *p < 0.05, **p < 0.01, Student’s t test. (**F**) Crystal structure of 1b showing its self-assembly in the solid state. Crystal data is available in Supplementary Tables S1–S8 and crystal packing in Supplementary Fig. S25.

Phenylethynylbenzyl (PEB)—disubstituted imidazolium and benzimidazolium were shown to self-assemble inside phospholipid membranes and form stable channels through π–π interactions (Fig. 1A)^13^. The transport of protons and ions through the open channels formed by compound **A.1** induced membrane perturbation, depolarization and bacterial death. Even if the PEB-disubstituted benzimidazolium salts **A.1** were very active on bacterial membranes, their potential as mitochondrial membrane perturbators was not further explored because of their high toxicity on red blood cells with an HC_50_ (concentration that kills 50% of red blood cells) of 0.15 mM^13,15^. The replacement of one PEB unit with a phenyl (**A.2**) or a methyl group (**A.3**) resulted in less toxic compounds (HC_50_ at 1 mM for the phenyl and 150 mM for the methyl substituent), as they form more compact aggregates in the solid state and probably in the membranes (Fig. 1A).

### Synthesis

PEB-Biguanidium chloride **1c** was synthesised by the cross-coupling of 4-bromobenzaldehyde with phenylacetylene followed by the reductive amination of the aldehyde with NaCNBH_3_. The biguanide was then formed by reacting the amine with dicyandiamide to afford PEB-biguanidium chloride **1c** with 30% yield. PEB-biguanidium chloride **2c** was obtained by hydrogenation of 4-(phenylethynyl)benzyl amine followed by the formation of the biguanidium chloride with 20% yield (Fig. 1C).

PEB-biguanidium **3** was synthesized by the cross-coupling of tert-Butyloxycarbonyl protected 4-iodoaniline with phenylacetylene followed by the deprotection of the amine and the formation of the biguanidium trifluoroacetate with dicyandiamide in a 24% yield. PEB-biguanidium **4** was synthesized by the hydrogenation of *tert*-butyl-(4-(phenylethynyl)phenyl)carbamate, deprotection of the amine and formation of the biguanidium trifluoroacetate with a 35% yield (Fig. 1D).

The counter-anions of PEB-Biguanidium chloride **1c** and **2c** were exchanged through the anion metastasis of chloride with either LiNTf_2_ (**a**) or LiOTf (**b**) in methanol in quantitative yield. The counter-anions of PEB-biguanidium **3** and **4** were exchanged by the same procedure with the addition of a deprotonation step with NaHCO_3_ before the ion exchange step.

### In vitro screening

The biguanidium salts possessing various counter-anions were first screened for growth inhibition of mouse pancreatic ductal adenocarcinoma PDAC cells NB508 and normal human fibroblasts IMR90 (Fig. 1E), at 5 µM. NB508 is an aggressive pro-metastatic pancreatic cancer cell line that we used previously to study the role of mitochondria in pancreatic cancer^16^. Almost all the tested compounds were able to inhibit the growth of cancer cells at this concentration, while no effect was observed for metformin and phenformin at the same concentration. The most important antiproliferative activities were observed for **1a** and **1b,** both showing a good selectivity towards cancer cells.

The toxicity of PEB-Biguanidiums salts was estimated through their hemolytic activity by incubating red blood cells (RBC) with the compounds for 1 h (See Supplementary Table S13 and Supplementary Fig. S29). A very low hemolytic activity (under 10%) was observed for all the compounds. As RBC are mainly composed of a plasma membrane enveloping hemoglobin, the low hemolysis observed indicates that PEB-Biguanidium salts are not disrupting RBC’s membranes, compared to the previously reported benzimidazolium salts^17,18^.

### Membrane activity

**Figure 2:**
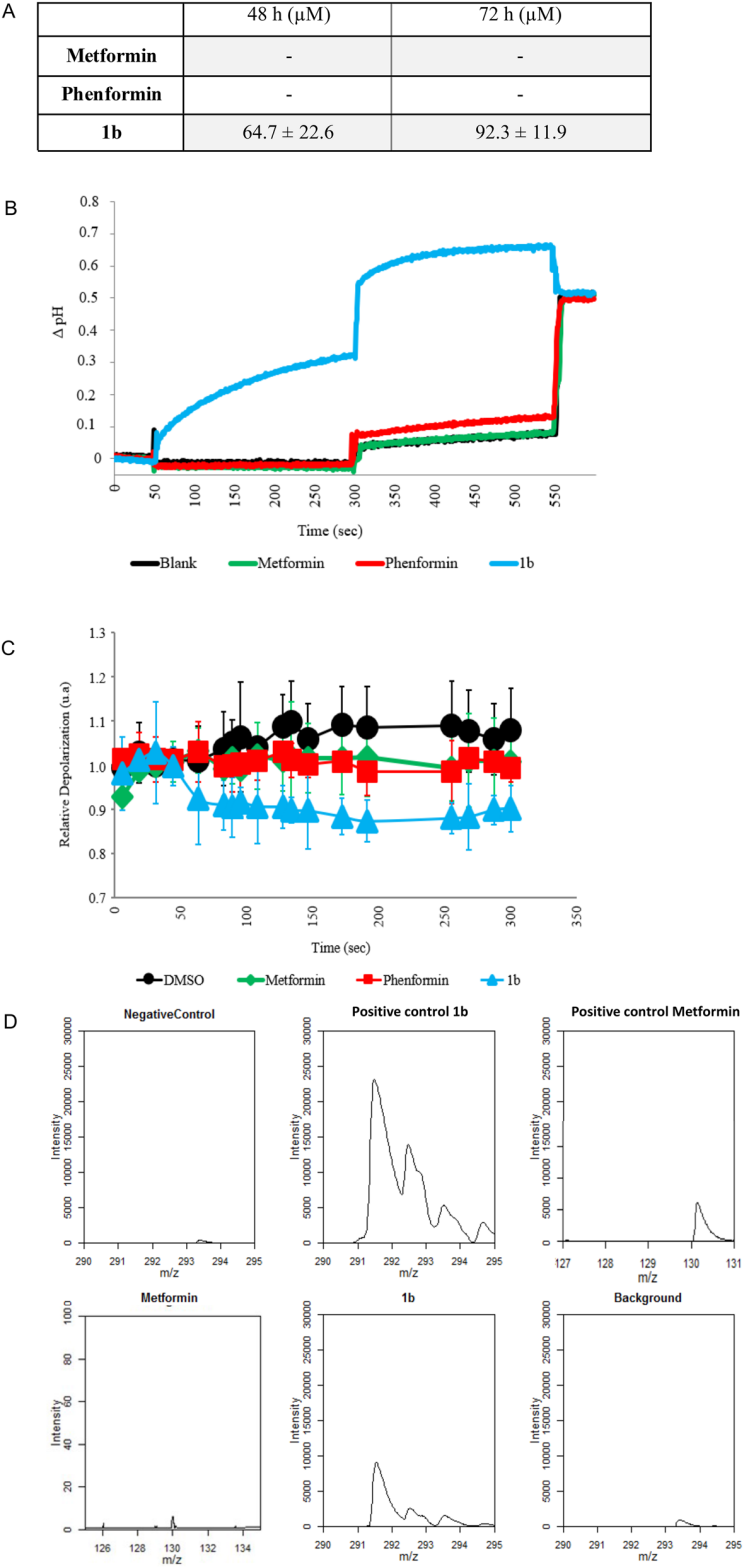
(**A**) Partition of compounds in an U-tube experiment. Concentration of biguanide on the *trans*-side of the U-tube at 48 h and 72 h at 25 °C, after addition of 250 µM of biguanide on the *cis*-side. (**B**) Variation in the internal pH of HPTS-containing EYPC liposomes. Intravesicular solution: 1 mM HPTS, 10 mM HEPES, and 100 mM NaCl, adjusted to pH = 7.4, and extravesicular solution: 10 mM HEPES and 100 mM NaCl, adjusted to pH = 7.4. Biguanidiums were injected after 50 s at 5 mM (50 mol% relative to the 10 mM EYPC concentration), a NaOH pulse was induced at 300 s, and the liposomes were lysed with Triton-X at 550 s. Each curve is the average of three independent measurements. (**C**) Fluorescence of safranin O in the EYPC liposomes. Biguanidium salts were injected at 5 mM (50 mol% relative to the 10 mM EYPC concentration) at 50 s. Each curve is the average of three independent measurements. (**D**) Mitochondrial penetration of compound **1b** and metformin. Mitochondrial isolation was performed according to the previously reported protocol^21^. For each experiment, an anti-HA IP was performed on KP4 cells expressing pMXs-3XHA-EGFP-OMP25 that were treated for 3 h with 15 μM metformin, 15 μM compound **1b** or vehicle. This shows mitochondrial penetration and accumulation of the drugs at 15 μM concentration after 3 h. As positive controls were used methanol solutions of metformin and **1b** at 15 μM, as negative control methanol and untreated mitochondria as vehicle.

Compound **1b** was the only one with good methanol and DMSO solubility even at high concentrations and was used for further investigation. The logP (see Supplementary Table S9 and Supplementary Fig. S26) of compound **1b** (0.4) is much higher than metformin (− 1.4)^19^ and phenformin (− 0.8)^20^, indicating its higher hydrophobicity and membrane permeability capacity. The ability of **1b** to penetrate and cross phospholipid membranes, compared to metformin and phenformin, was studied by U-tube experiments, where two aqueous phases are separated by a bulk hydrophobic solvent such as chloroform, to mimic a bilayer membrane (see Supplementary Tables S10–11 and Supplementary Fig. S27)*.* A compound able to penetrate and cross a phospholipid bilayer is able in these conditions to partition into water and chloroform. A concentration of 250 µM of each biguanide was added to the *cis*-aqueous side and the concentration of the *trans*-side was measured after 48 h and 72 h. Compound **1b** was able to partition rapidly into the chloroform and cross to the *trans*-aqueous side, while only traces of metformin and phenformin were measured after 72 h (Fig. 2A).

Phospholipid vesicles can be used as models for studying membrane insertion and permeabilization^22^. Using an anion-selective probe in synthetic phospholipid liposomes, anion transport can be monitored by fluorescence. Despite its structural similarity to the PEB-substituted benzimidazolium synthetic transporters and ability to penetrate phospholipid membranes as showed by the U-tube test, compound **1b** did not transport chloride across the phospholipid membrane of egg yolk phosphatidylcholine large unilamellar vesicles (EYPC-LUVs) (see supporting information and Supplementary Fig. S28). As it can be observed in the solid-state structure obtained from chloroform, compound **1b** does not form channel-like supramolecular architectures but self-assembles into herringbone-shaped interlocked dimers that do not seem to possess chloride transport properties (Fig. 1F). However, when using EYPC-LUVs containing 8-Hydroxypyrene-1,3,6-trisulfonic acid trisodium salt (HPTS) as pH-sensitive probe, a basification of the internal vesicular pH was observed when compound **1b** was added (Fig. 2B). This basification was further enhanced with the addition of a base pulse to the external solution at 300 s, indicating either a transport of protons to the extravesicular solution, or the transport of hydroxyls to the intravesicular solution. This was also reported for alkylbiguanidium salts and was described as an electrogenic process, *i.e.* the transmembrane transport of an ion without the balance of the charge through the movement of another ion, which usually results in a charge transfer^16^. Compound **1b**, like alkylbiguanidium salts, was not able to transport chloride, so the electroneutral hypothesis of a H^+^/Cl^−^ antiport or OH^−^/Cl^−^ symport can be rejected, and the hypothesis of the electrogenic transport mechanism can be suggested. Metformin and phenformin showed no membrane activity in these experiments. The transport of H^+^/OH^−^ ions across the membrane is an electrogenic mechanism that usually causes the depolarization of the electrochemical gradient across phospholipid membranes. When EYPC-LUVs liposomes containing intravesicular K^+^ ions are bathing in an extravesicular solution containing Na^+^ ions a small membrane potential is generated at their membranes, and the fluorescence of Safranin-O can be used as a membrane potential probe^23^. The addition of **1b** to the external solution of these Safranin-O-containing liposomes resulted in a decrease of the Safranin-O’s fluorescence intensity, indicating a depolarization of the membrane, while again metformin or phenformin did not show this activity (Fig. 2C).

Mitochondrial membrane potential together with the proton gradient are responsible for ATP production^24^ and maintenance of cellular health and function. Compounds able to penetrate mitochondria and alter their membrane potential are usually used as chemotherapeutics. Compound **1b**, but not metformin, was able to efficiently penetrate mitochondria after a short incubation of cells with 15 µM concentration of each drug (Fig. 2D). This indicates that compound **1b** easily diffuses into mitochondria unlike metformin that requires a specialized transporter^25^. These experiments altogether indicate that **1b** is able to penetrate phospholipid and mitochondrial membranes and alter their potential.

### Antiproliferative activity in vitro and mechanism of action

**Figure 3:**
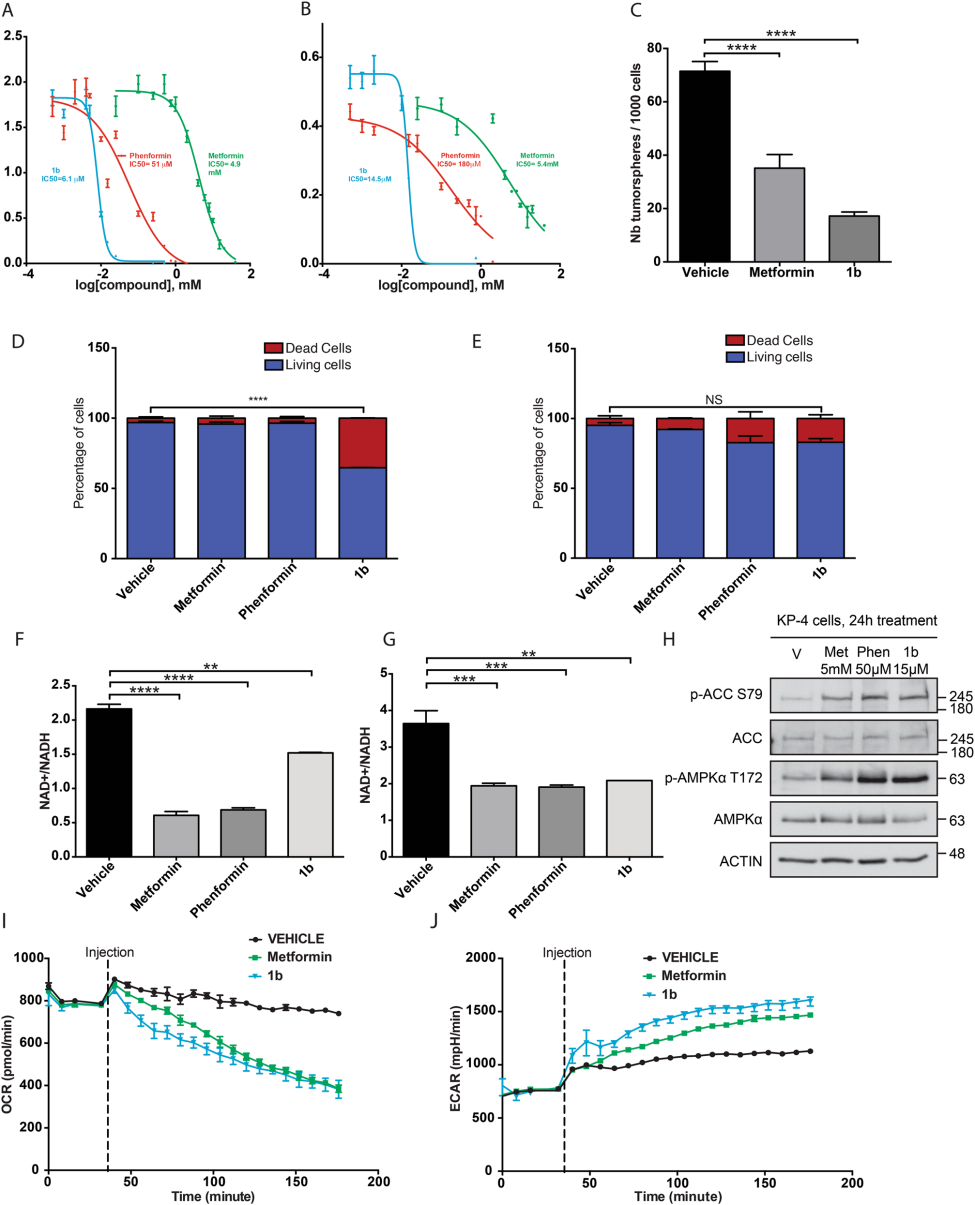
Effects of biguanides on cell proliferation and viability in pancreatic cancer cells. (**A**) IC_50_ of metformin, phenformin and compound **1b** performed in vitro over 3 days on KP4 cells. (**B**) IC_50_ of metformin, phenformin and compound **1b** performed in vitro over 3 days on Panc1 cells. (**C**) Effect of 24 h treatment with metformin (1 mM) or compound **1b** (5 µM) on the formation of tumor spheres in AH375 cells grown in suspension (mouse pancreatic ductal adenocarcinoma). ****p ≤ 0.0001 (ANOVA). (**D**,**E**) Proportion of living and dead cells after 24 h treatment, with either metformin (5 mM), Phenformin (50 µM), compound **1b** (15 µM) or vehicle in KP4 cells **** p ≤ 0.0001 (ANOVA) (**D**) and HPNE-hTERT cells (**E**). (**F**) Effect of treatment for 6 h of PSN1 cells with metformin (10 mM), phenformin (100 µM) or compound **1b** (25 µM) on NAD^+^/NADH ratio. **p ≤ 0.01, ****p ≤ 0.0001 (ANOVA). (**G**) Effect of treatment for 18 h of KP4 cells with metformin (5 mM), phenformin (50 µM) or compound **1b** (15 µM) on NAD + /NADH ratio. **p ≤ 0.01, ***p ≤ 0.001 (ANOVA). (**H**) Effect of treatment for 24 h of KP4 cells with metformin (5 mM), phenformin (50 µM) or compound **1b** (15 µM) on phosphorylation levels of AMPK T172 and ACC S79. (**I**) Effect of treatment of KP4 cells with metformin (10 mM) or compound **1b** (15 µM) on oxygen consumption rate (OCR) measured by Seahorse analysis. (**J**) Effect of treatment of KP4 cells with metformin (10 mM) or compound **1b** (15 µM) on ECAR (extracellular acidification rate) measured by Seahorse analysis.

In order to compare the antiproliferative/anticancer activity of compound **1b** to that of the most commonly studied biguanides-derivatives metformin and phenformin, we performed growth assays with human PDAC cell lines and normal immortalized pancreatic epithelial cells (HPNE) over 72 hours in the presence of various concentrations of these compounds. The results showed that both in KP4 cells and in Panc1 cells (Fig. 3A,B and Supplementary Table S12), metformin has a half maximal inhibitory concentration (IC_50_) of about 5 mM. Phenformin, with an hydrophobic phenyl group, has as lower IC_50_ of 51 µM in KP4 and 180 µM in Panc1 cells. Compound **1b** showed much lower IC_50_ of 6.1 µM in KP4 cells and 15 µM in Panc1 cells, 100–800 fold lower than metformin and 8–12 fold lower than phenformin. The IC_50_ of most of the synthetic biguanides in HPNE cells was higher than for cancer cells (Supplementary Table S12).

We have recently shown that a short treatment of 24 h with metformin of the mouse PDAC cell line AH375 grown adherent in 2D decreases the ability of this cell line to subsequently grow as spheres in suspension^16^. Here again compound **1b** shows a better biological activity than metformin (Fig. 3C) as it decreases by 75% the number of AH375 spheres when treated for 24 h with 5 µM of it, while metformin at 1 mM results in a less marked decrease of 50%. In agreement with its higher ability to inhibit cancer cells, we observed that compound **1b** induces a significant increase (~ 20%) of the fraction of dead KP4 cells (Fig. 3D) treated at 15 µM for 24 h while metformin (5 mM) and phenformin (50 µM) showed no significant effects. In contrast with its effect in PDAC KP4 cells, compound **1b** did not increase the fraction of dead cells in HPNE cells in the same conditions (Fig. 3E), suggesting selectivity towards cancer cells.

Metformin decreases the NAD^+^/NADH ratio through inhibition of respiratory complex I^26^. To better characterize the molecular mechanism of action of compound **1b**, we measured its effect on the NAD^+^/NADH ratio in comparison with other biguanides using the concentrations at which these compounds inhibit cell proliferation. In agreement, both in pancreatic cancer PSN1 cells (Fig. 3F) and KP4 cells (Fig. 3G) compound **1b** at 15 µM decreased NAD^+^/NADH ratio by 25% and 40% respectively, as did phenformin (50 µM) and metformin (5 mM). These results suggest that, as metformin and phenformin do, compound **1b** is able to inhibit mitochondrial respiration, but at a much lower concentration. In addition, as it is broadly reported for metformin and phenformin^27^, compound **1b** induced AMPK activation as assessed by measuring AMPK phosphorylation on T172 and that of the AMPK-target acetyl-CoA carboxylase (ACC) (Fig. 3H).

Next, we used the Seahorse analyser to quantify the ability of compound **1b** to affect oxygen consumption rate (OCR) in KP4 cells. We were able to determine that compound **1b** inhibited OCR at 15 µM, similarly to metformin at 5 mM (Fig. 3I). Inhibition of OCR by metformin was simultaneously associated with an increase of the ECAR (Extracellular Acidification Rate, a measure for glycolysis), which was also observed with compound **1b** (Fig. 3J). These data suggest that the anticancer activity of compound **1b** is due at least in part to the inhibition of mitochondrial respiration, similar to metformin, but at much lower concentration.

**Figure 4:**
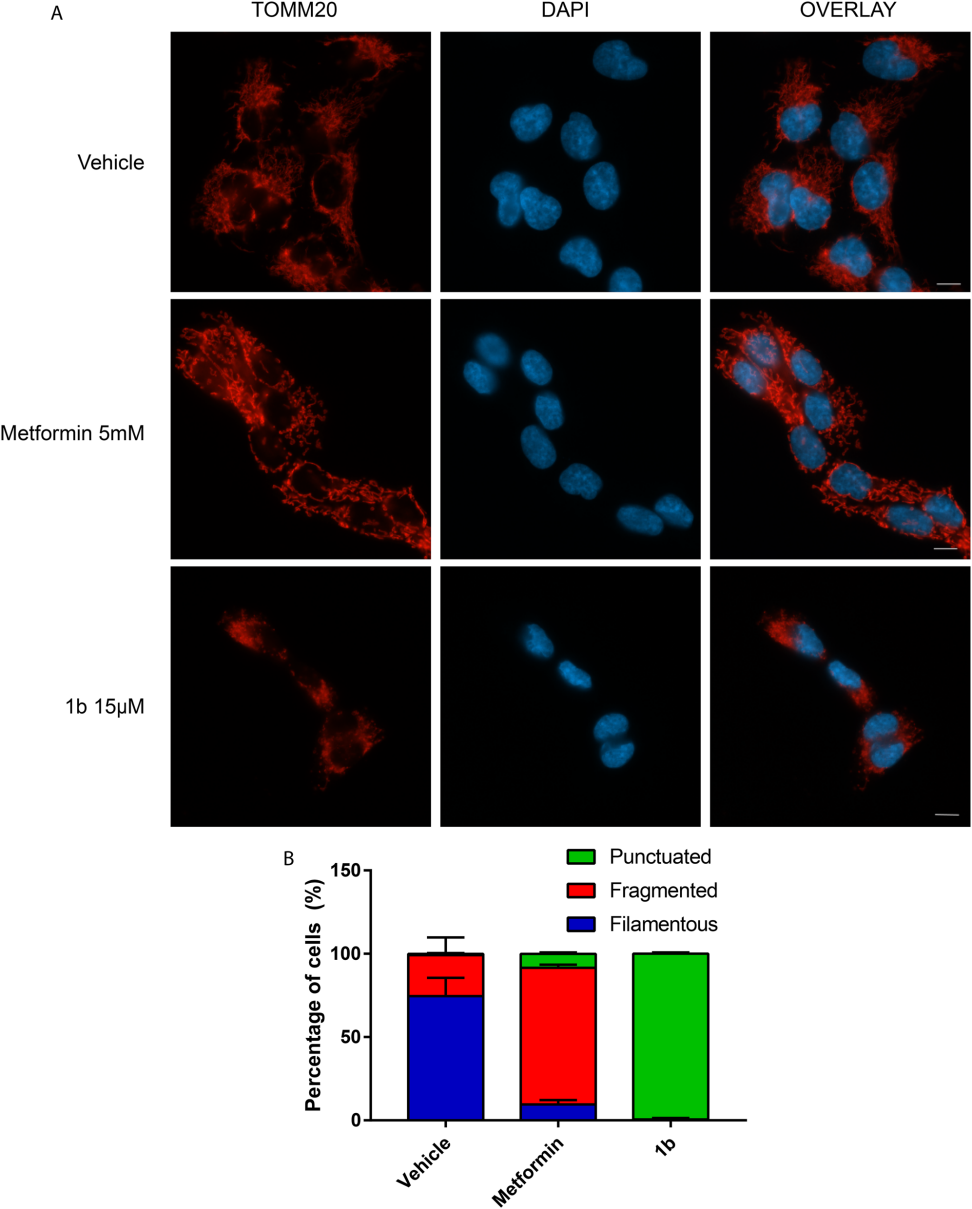
Effects of biguanides on mitochondrial morphology. (**A**) Mitochondrial morphology in KP4 cells 24 h following the indicated treatments as visualised by anti-TOMM20 immunofluorescence. Scale bar = 10 µM. (**B**) Quantification of the percentage of cells exhibiting filamentous, fragmented or punctuated mitochondria following indicated treatments. The data represents the mean of 2 biological replicates and for each replicate three counts of 50 cells were done. n = 300 cells per treatment. Data was analyzed with one way ANOVA followed by Tukey HSD test.

The action of biguanides in mitochondria can lead to morphological alterations that include disorganisation of the cristae^28^. To further characterize the metformin-like activity of compound 1b, we visualized mitochondria in cells treated with this compound and metformin. Both compound **1b** and metformin led to a loss of the filamentous mitochondrial network but compound **1b** had a bigger impact in mitochondrial morphology leading to a punctuated pattern typical of fragmented mitochondria (Fig. 4A,B). Together these data show that compound **1b** functions as metformin or phenformin but with a much higher activity, presumably due to its intrinsic chemical characteristics and membrane activity.

### Antiproliferative activity in vivo

**Figure 5:**
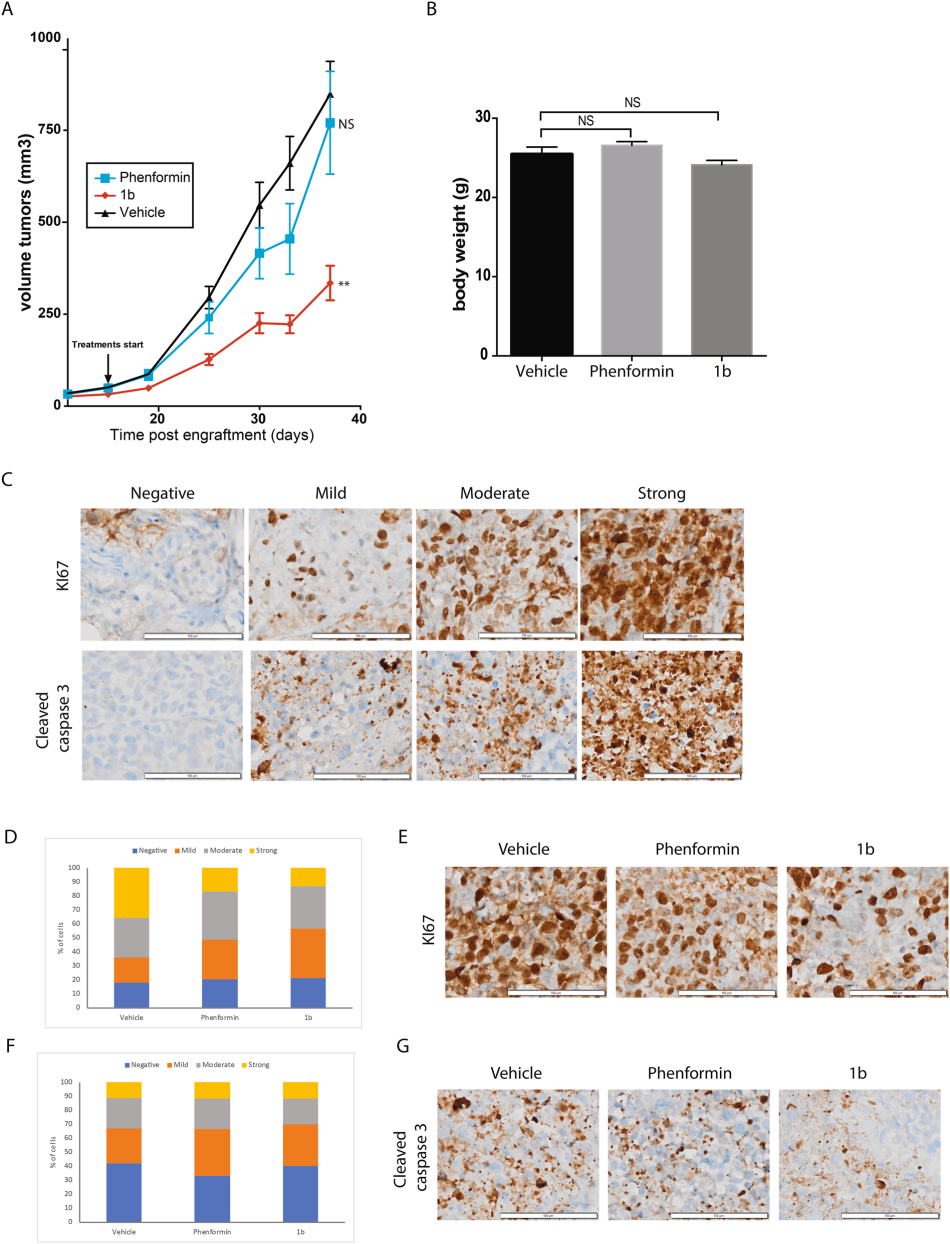
Progression of the volume of KP4 cells sub-cutaneous xenografts performed in nude mice over 37 days. (**A**) Treatments with phenformin or compound **1b** (both at 50 mg/kg/day, 5 days a week) or vehicle were started 11 days post engraftment. **p ≤ 0.01, NS: not significative (ANOVA). (**B**) Body weight of mice seven days after treatment. NS: not significative (ANOVA) (**C**) Scale of staining intensity calculated with the immunoreactivity scoring method for KI67 (top) and cleaved caspase 3 (bottom) staining. Representative images shown. Scale bar = 100 µm. (**D**,**F**) Analysis of KI67 (**D**) and cleaved caspase 3 (**F**) staining on tumor sections from mice treated with vehicle, phenformin or **1b** (both at 50 mg/kg). n = 3. Results are shown in graphics comparing the percentage of cells stained in each category and the intensity of staining. (**E**,**G**) Images of the most representative phenotypes for KI67 staining (**E**) and cleaved caspase 3 (**G**).

To determine whether compound **1b** is also more efficient to phenformin in vivo, mice were engrafted with subcutaneous KP4 cells tumors. After randomization, mice received either phenformin (n = 6 mice, 2 tumors per mouse), compound **1b** (n = 17 mice, 2 tumors per mouse) both at (50 mg/kg/day IP, 5 day/week) or vehicle (n = 10 mice, 2 tumors per mouse). This dose was chosen from previous work on biguanides to treat mice with pancreatic cancer^29^. Compound **1b** significantly reduced tumor volume (Fig. 5A) and did not affect body weight (Fig. 5B), while phenformin administered at the same dose had only modest or non-significant effects. Together our data show that in vivo, compound **1b** is well tolerated and more effective than the most active biguanide used currently in humans.

To assess the mechanism of the antitumor activity, we stained tumor sections obtained from animals after the treatment protocol for proliferation marker KI67 and apoptosis marker cleaved caspase 3, and quantified the data according to the scoring key in Fig. 5C. Compound **1b**, and to a smaller extent phenformin, reduced KI67 staining in tumors (Fig. 5D). However, there were no significant differences in staining for cleaved caspase 3 (Fig. 5E). This indicates that biguanides do not trigger apoptosis in tumors in vivo and the observed reduction in tumor cell viability in vitro must be related to other cell death pathways that can be activated by biguanides^30^.

## Conclusion

In conclusion, by screening a small library of phenylethynylbenzyl-modified metformin analogs, we identified a compound able to penetrate mitochondrial membranes, 1000-fold more active than metformin. Compound **1b** selectively reduced proliferation and viability of PDAC cells (KP4), at least in part by the inhibition of mitochondrial respiration, in a similar way to metformin but at much lower concentration. Compound **1b** is more active than metformin and phenformin to inhibit the proliferation of PDAC cells in vivo, as it significantly inhibits the growth of pancreatic cancer xenografts in mice. We demonstrate herein that the anticancer properties of biguanides can be improved by chemical modifications. The new PEB-Biguanidium compound is a first-class lead compound that can be used to target mitochondria in pancreatic cancer and potentially other cancers.

## Methods

All chemicals were purchased from Aldrich Chemicals in their highest purity and used without further purification. Deuterated dimethylsulfoxide (DMSO-*d*^6^) and deuterated chloroform (CDCl_3_) were purchased from CDN Isotopes. NMR spectra were recorded on Bruker advance 400 and 100 spectrometers. Coupling constants are given in hertz (Hz) and chemical shifts are given in parts per million (ppm, δ) measured relative to the residual solvent (the multiplicity of the signals are given as s: singlet, d: doublet, t: triplet, and m: multiplet). High-resolution mass spectra (HRMS) were recorded on a TSQ Quantum Ultra (Thermo Scientific) triple quadrupole with accurate mass option instrument (Université de Montréal Mass Spectrometry Facility). MS experiences were performed using an UltrafleXtreme MALDI TOF/TOF mass spectrometer equipped with a SmartBeam II Nd:Yag/355 nm laser operating at 1 kHz and providing a laser focus down to 20 μm in diameter (Bruker Daltonics, Billerica, MA). The data acquisition for MS was performed in positive ion mode using the linear geometry with flexControl 3.4 (Bruker Daltonics, Billerica, MA). Acceleration voltage was set to + 25 kV and all other instrumental parameters (delayed extraction parameters, source voltages, detector gain, laser energy, etc.) were optimized for maximum S/N for the drug compounds. l-α-Phosphatidylcholine was purchased from Avanti Polar Lipids. Transport and depolarization studies as well as absorbance measurements were performed on a Varian Cary Eclipse fluorescence spectrophotometer. The hemolysis assays were performed on a Fluostar Optima plate reader. Single crystals of C_18_H_18_F_3_N_5_O_3_S were obtained from chloroform. A suitable crystal was selected and analysed on a Bruker Venture Metaljet diffractometer. Crystal violet retention assay was used for cell growth assays and IC_50_ were determined using Prism (GraphPad). NAD/NADH quantitation colorimetric kit (#K337-100) from Biovision was used according to manufacturer’s instructions. ECAR (extracellular acidification rate) and OCR of KP4 cells (oxygen consumption rate) were measured on a Seahorse XF-24 (Agilent).

Detailed synthetic procedures, NMR and MS spectra, single crystal X-rays diffraction data, log P measurement, U-tube experiments, lucigenin, HPTS and safranin O assays, mitochondrial permeation and accumulation, hemolytic activity, NAD/NADH quantification, animal experiments and immunoblots are given in the Supplemental Information.

All experiments were performed in accordance to the rules of the in vivo ethical committee of University of Montreal (CDEA approval #17-103), in compliance with the ARRIVE guidelines.

## Supplementary Information

Supplementary Information.
