# Supplementary material for: Phenylethynylbenzyl-modified biguanides inhibit pancreatic cancer tumor growth
Source: Sci Rep. 2021 May 10;11:9854. doi: 10.1038/s41598-021-87993-3 (PMC8110578; doi:10.1038/s41598-021-87993-3)
Supplement: Supplementary file 1 — Supplementary Information. [file 41598_2021_87993_MOESM1_ESM.docx]

**Supporting Information**

**Phenylethynylbenzyl-Modified Biguanides Inhibit Pancreatic Cancer Tumor Growth**

Audrey Hébert^[a]❖^, Maxime Parisotto^[a]❖^, Marie-Camille Rowell^[b]^, Alexandra Doré^[a]^, Ana Fernandez^[b]^, Guillaume Lefrancois^[a]^, Paloma Kalegari^[b],^ Gerardo Ferbeyre*^[b]^ and Andreea R. Schmitzer*^[a]^

*^[a]^ Département de Chimie - Faculté des Arts et des Sciences, Université de Montréal, 2900 Edouard Montpetit, CP 6128 Succursale Centre-Ville, Montréal, H3C3J7, Québec, Canada. ^[b]^ Département de Biochimie et Médecine Moléculaire - Faculté de Médecine, Université de Montréal.*

^❖^*Contributed equally to this work*

Corresponding author: [*ar.schmitzer@umontreal.ca](mailto:*ar.schmitzer@umontreal.ca) or [g.ferbeyre@umontreal.ca](mailto:g.ferbeyre@umontreal.ca)

**Table of contents**

[1. Synthesis and Characterization of the compounds 2](#_Toc49759486)

[Synthetic procedures 2](#_Toc49759487)

[NMR Spectra of the synthesized compounds 11](#_Toc49759488)

[2. Single crystal X-ray diffraction 23](#_Toc49759489)

[3. Measurement of the hydrophobicity of compound 1b 37](#_Toc49759490)

[4. U-Tube experiments 38](#_Toc49759491)

[5. Lucigenin assay 40](#_Toc49759492)

[6. HPTS assay 41](#_Toc49759493)

[7. Safranin O assay 41](#_Toc49759494)

[8. Mitochondrial permeation and accumulation 41](#_Toc49759495)

[10. Hemolytic activity 43](#_Toc49759496)

[11. NAD/NADH quantification 45](#_Toc49759497)

[13. Animal experiments 45](#_Toc49759498)

[14. Immunoblots 45](#_Toc49759499)

# Synthesis and Characterization of the compounds

##

## Synthetic procedures

***4-(phenylethynyl)benzaldehyde.*** 4-Bromobenzaldehyde (4.00 g, 21.6 mmol), PdCl_2_(PPh_3_)_2_ (0.091g, 0.13 mmol), CuI (0.082g, 0.43 mmol) and triphenylphosphine (0.113g, 0.43 mmol) were dissolved in a mixture of THF (100 ml) and triethylamine (22.4 ml, 173 mmol). Phenylacetylene (2.85 ml, 25.9 mmol) was added slowly and the mixture was heated to 70°C overnight. The mixture was cooled to room temperature, filtered and washed with THF and concentrated in vacuo. The residue was then purified by flash chromatography (EtOAc/hexane gradient) to afford 4-(phenylethynyl)benzaldehyde (4.42 g, 21.6 mmol) as a crystalline solid (quantitative yield %)

^1^H NMR (400 MHz, CDCl_3_, 25 °C, TMS): δ = 7.40 (m, 3 H), 7.58 (m, 2H), 7.70 (d, *J* = 8.0 Hz, 2H), 7.90 (d, *J* = 4.0 Hz, 2H), 10.05 (s, 1H)

^13^C NMR (70 MHz, CDCl_3_, 25 °C, TMS_,_): δ = 89.1, 93.4, 122.1, 128.6, 129.3, 129.9, 130.1, 132.1, 132.5, 135.9, 192.9

HRMS (120.0V, ES+): *m*/*z* (%) = 207.0813 ([C_15_H_11_N_5_]+H)^+^, 208.0843 (M + H)^+^.

***4-(phenylethynyl)benzylamine.*** 4-(phenylethynyl)benzaldehyde (4.42g, 21.4 mmol) and sodium cyanoborohydride (4.04, 64.3 mmol) were dissolved in a solvent mixture of EtOH saturated with NH_4_OAc and NH_4_OH 5:2 (30 mM), and reaction was heated at 80°C overnight. EtOH was evaporated under reduced pressure, resulting mix was extracted with DCM and washed with NaHCO_3_. The mixture was concentrated in vacuo and purified by flash chromatography (DCM/MeOH gradient) to afford 4-(phenylethynyl)benzylamine (1.02 g, 4.9 mmol) as a white solid. (20% yield)

^1^H NMR (400 MHz, DMSO-d_6_, 25 °C, TMS): δ = 3.77 (s, 2H), 7.42 (m, 6H), 7.52 (d, *J* = 8.0 Hz, 2H), 7.55 (m, 3H)

^13^C NMR (70 MHz, DMSO-d_6_, 25 °C, TMS): δ = 42.4, 89.8, 90.4, 122.5, 122.8, 129.3, 129.4, 129.7, 131.9, 132.0, 135.1

HRMS (120.0V, ES+): *m*/*z* (%) = 208.1126 ([C_15_H_14_N_1_]+H)^+^, 209.1163 (M + H)^+^.

***4-(phenylethynyl)benzylbiguanide chloride salt (1).*** 4-(phenylethynyl)benzylamine (1.00 g, 4.82 mmol), dicyandiamide (0.811 g, 9.64 mmol) and trimethylsilylchloride (2.45 ml, 19.3 mmol) were dissolved in anhydrous THF (23.8ml, 202 mM) in a sealed tube. The mixture was heated to 145°C for 1h. The mixture was filtered and washed with THF, and residue was purified by TLC prep (DCM:MeOH 9:1) to afford 4-(phenylethyl)benzylbiguanide as its chloride salt (0.460g, 1.40 mmol, 30% yield)

^1^H NMR (400 MHz, DMSO-d_6_, 25 °C, TMS): δ = 4.41 (d, 2H), 7.00 (s, 6H), 7.37 (d, *J* = 4.0 Hz 3H), 7.44 (m, 4H), 7.55 (m, 5H)

^13^C NMR (70 MHz, DMSO-d_6_, 25 °C, TMS): δ = 44.5, 89.6, 121.4, 122.7, 128.0, 129.2, 131.8, 158.9, 160.8

HRMS (120.0V, ES+): *m*/*z* (%) = 292.1567 ([C_17_H_22_N_5_]+H)^+^, 293.1590 (M + H)^+^.

Purity was assessed by HPLC, > 99%

***4-(phenylethyl)benzylamine.*** 4-(phenylethynyl)benzylamine (500 mg, 2.41 mmol) and palladium on carbon 10 wt.% (0.51g, 0.48 mmol) were mixed in 200 ml of a EtOH:AcOEt 1:1 mixture under nitrogen. Reaction was put in a H_2_ atmosphere and heated to 60 °C for 2h. After purging with nitrogen, reaction was filtered on a celite pad and evaporated under reduced pressure to afford 4-(phenylethyl)benzylamine (500 mg, 2.41 mmol) as a white solid (quantitative yield)

^1^H NMR (400 MHz, DMSO-d_6_, 25 °C, TMS): δ = 2.87 (s, 4H), 3.70 (s, 2H), 7.18 (m, 3H), 7.27 (m, 8H)

^13^C NMR (70 MHz, DMSO-d_6_, 25 °C, TMS): δ = 37.2, 37.6, 45.5, 126.3, 127.6, 128.6, 128.7, 128.9, 140.0, 141.2, 142.0

LRMS (120.0V, ES+): *m*/*z* (%) = 212.14 ([C_15_H_19_N_1_]+H)^+^

***4-(phenylethyl)benzylbiguanide chloride salt (2).*** 4-(phenylethyl)benzylamine (0.273 g, 1.29 mmol), dicyandiamide (0.217 g, 2.58 mmol) and trimethylsilylchloride (0.655 ml, 5.16 mmol) were dissolved in anhydrous THF (6.38 ml, 202 mM) in a sealed tube. The mixture was heated to 145°C for 1h. The mixture was filtered and washed with THF, and residue was purified by TLC prep (DCM:MeOH 9:1) to afford 4-(phenylethyl)benzylbiguanide as its chloride salt (72 mg, 0.24 mmol, 20% yield)

1H NMR (400 MHz, DMSO-d_6_, 25 °C, TMS): δ = 2.87 (s, 4H), 4.30 (d, *J* = 4.0 Hz, 2H), 6.92 (s, 5H), 7.24 (m, 10H), 7.64 (s, 1H)

^13^C NMR (70 MHz, DMSO-d_6_, 25 °C, TMS): δ = 37.2, 37.5, 42.5, 44.5, 126.3, 127.7, 128.7, 128.8, 129.0, 129.4, 132.0, 140.7, 141.8, 141.9, 142.3, 159.0, 160.3

HRMS (120.0V, ES+): *m*/*z* (%) = 296.1876 ([C_17_H_22_N_5_]+H)^+^

Purity was assessed by HPLC, 95%

***tert-butyl (4-(phenylethynyl)phenyl)carbamate.*** Iodoaniline (3.0 g, 14.0 mmol) and di-*tert*-butyl dicarbonate (3.0 g, 14.0 mmol) were mixed in THF at room temperature overnight. PdCl_2_(PPh_3_)_2_ (0.058g, 0.082 mmol), CuI (0.047g, 0.28 mmol) and triphenylphosphine (0.047g, 0.28 mmol) and triethylamine (13.5 ml, 112.0 mmol) were added to the mix, and phenylacetylene (1.5 ml, 14.0 mmol) was added slowly. Reaction was heated to 60°C ovenight. The mixture was cooled to room temperature, filtered and concentrated in vacuo. The residue was then purified by flash chromatography (EtOAc/hexane gradient) to afford *tert*-butyl (4-(phenylethynyl)phenyl)carbamate (1.39 g, 4.7 mmol, 78% yield)

^1^H NMR (400 MHz, CDCl_3_, 25 °C, TMS):δ = 1.55 (s, 9 H), 6.57 (s, 1H), 7.37 (m, 5H), 7.49 (d, *J* = 8.0 Hz, 2H), 7.54 (m, 2H)

^13^C NMR (70 MHz, CDCl_3_, 25 °C, TMS): δ = 27.4, 28.3, 89.3, 117.5, 118.03, 123.43, 128.0, 128.3, 131.5, 132.45, 138.4, 146.75

LRMS (120.0V, ES+): *m*/*z* (%) = 294.38 ([C_19_H_19_NO_2_]+H)^+^

***4-(phenylethynyl)aniline trifluoroacetic salt.*** *tert*-butyl (4-(phenylethynyl)phenyl)carbamate (1.39g, 4.7 mmol) and trifluoroacetic acid (5 eq.) were stirred in DCM at 60 °C for 2h. Solvent was evaporated under reduced pressure and reaction was purified on a silica column (DCM:MeOH gradient) to afford 4-(phenylethynyl)aniline trifluoroacetic salt (1.44 g, 4.7 mmol, quantitative yield)

^1^H NMR (400 MHz, DMSO-d_6_, 25 °C, TMS): δ = 4.15 (s, 2 H), 6.57 (d, *J* = 8.0 Hz, 2H), 7.27 (m, 5H), 7.77 (d, *J* = 12.0 Hz, 2H)

^13^C NMR (70 MHz, DMSO-d_6_, 25 °C, TMS): δ = 91.6, 114.1, 123.8, 128.0, 129.1, 131.2, 133.2, 150.0

LRMS (120.0V, ES+): *m*/*z* (%) = 212.11 ([C_14_H_11_N_1_]+H)^+^

***(4-(phenylethynylphenyl)biguanide trifluoroacetic salt (3).*** 4-(phenylethynyl)aniline trifluoroacetic salt (0.350 g, 1.19 mmol), dicyandiamide (0.20 g, 2.37 mmol) and trimethylsilylchloride (0.602 ml, 4.74 mmol) were dissolved in anhydrous THF (5.86 ml, 202 mM) in a sealed tube. The mixture was heated to 145°C for 1h. The mixture was filtered and washed with THF, and residue was triturated with EtOH to afford (4-(phenylethynylphenyl)biguanide as its trifluoroacetic acid salt (0.18 g, 0.31 mmol, 26% yield)

^1^H NMR (400 MHz, DMSO-d_6_, 25 °C, TMS): δ = 7.08 (s, 3 H), 7.27 (m, 6H), 7.45 (s, 3H), 7.52 (d, *J* = 8.0 Hz, 2H), 7.99 (d, *J* = 12.0 Hz, 2H)

^13^C NMR (70 MHz, DMSO-d_6_, 25 °C, TMS): δ = 44.9, 119.5, 126.9, 128.7, 130.0, 131.0, 135.9, 156.0, 163.3

MS (120.0V, ES+): *m*/*z* (%) = 296.1508 ([C_16_H_15_N_5_]+H+NH_4_)^+^

Purity was assessed by HPLC, 95%

***tert-butyl (4-phenethylphenyl)carbamate.*** *tert*-butyl (4-(phenylethynyl)phenyl)carbamate (0.35g, 1.19 mmol) and palladium on carbon 10 wt.% (0.25 g, 0.239 mmol) were mixed in 200 ml of a EtOH:AcOEt 1:1 mixture under nitrogen. Reaction was put in a H_2_ atmosphere and heated to 60 °C for 2h. After purging with nitrogen, reaction was filtered on a celite pad and evaporated under reduced pressure to afford *tert*-butyl (4-phenethylphenyl)carbamate (0.35g, 1.9 mmol) as a white solid (quantitative yield)

^1^H NMR (400 MHz, CDCl_3_, 25 °C, TMS): δ = 1.53 (s, 9H),2.89 (s, 4H), 6.42 (s, 1H), 7.11 (d, *J* = 8.0 Hz, 3H), 7.19 (m, 4H) ,7.28 (m, 2H)

^13^C NMR (70 MHz, CDCl_3_, 25 °C, TMS): δ = 28.5, 37.3, 38.1, 58.5, 80.4, 118.7, 126.0, 128.3, 128.6, 129.0, 136.3, 136.6, 141.9

LRMS (120.0V, ES+): *m*/*z* (%) = 298.52 ([C_19_H_23_NO_2_]+H)^+^

***4-(phenylethylphenyl)aniline trifluoroacetic salt.*** *tert*-butyl (4-(phenylethyl)phenyl)carbamate (0.2 g, 0.63 mmol) and trifluoroacetic acid (5 eq.) were stirred in DCM at 60 °C for 2h. Solvent was evaporated under reduced pressure and reaction was purified on a silica column (DCM:MeOH gradient) to afford 4-(phenylethylphenyl)aniline trifluoroacetic salt (1.44 g, 4.7 mmol, quantitative yield)

^1^H NMR (400 MHz, DMSO-d_6_, 25 °C, TMS): δ = 2.88 (s, 4 H), 7.07 (s, 1H), 7.18 (m, 6H), 7.27 (m, 5H),

^13^C NMR (70 MHz, DMSO-d_6_, 25 °C, TMS): δ = 36.6, 37.4, 121.9, 126.3, 128.7, 128.9, 130.0, 133.0, 141.7

LRMS (120.0V, ES+): *m*/*z* (%) = 198.50 ([C_14_H_15_N_1_]+H)^+^

***(4-(phenylethylphenyl)biguanide trifluoroacetic acid salt (4).*** 4-(phenylethylphenyl)aniline trifluoroacetic salt (0.350 g, 1.17 mmol), dicyandiamide (0.20 g, 2.34 mmol) and trimethylsilylchloride (0.594 ml, 4.68 mmol) were dissolved in anhydrous THF (5.86 ml, 202 mM) in a sealed tube. The mixture was heated to 145°C for 1h. The mixture was filtered and washed with THF, and residue was triturated with EtOH to afford (4-(phenylethylphenyl)biguanide as its trifluoroacetic acid salt (0.150 g, 0.41 mmol, 35%)

^1^H NMR (400 MHz, DMSO-d_6_, 25 °C, TMS): δ = 2.86 (s, 4 H), 7.23 (m, 10H), 7.45 (s, 6H), 8.70 (s, 1H)

^13^C NMR (70 MHz, DMSO-d_6_, 25 °C, TMS): δ = 36.9, 37.5, 122.2, 126.3, 128.7, 128.9, 129.2, 136.9, 141.9, 155.9, 161.4

MS (120.0V, ES+): *m*/*z* (%) = 282.1721 ([C_16_H_19_N_5_]+H)^+^

Purity was assessed by HPLC, > 99%

***Anion exchange: general procedure.***

PEB-biguanidium **3** and **4** as their trifluoroacetic salt were dissolved in methanol and deprotonated with NaHCO_3_ (4 equivalents) for 3 hours at room temperature. The mixture was concentrated in vacuo and the residue was triturated with EtOAc. The precipitate was filtered and washed to afford deprotonated PEB-biguanide **3** and **4** in quantitative yield.

PEB-biguanidium **1** and **2** as their chloride salt and bis(trifluoromethane)sulfonamide lithium salt (LiNTf_2_, 2.5 equivalents) or lithium trifluoromethanesulfonate (LiOTf, 2.5 eq) were dissolved in methanol and stirred overnight at room temperature. PEB-biguanidium **3** and **4** were prepared following the same procedure with the addition of 2 equivalents of HCl to the reaction. The mixture was concentrated in vacuo and the residue was triturated in EtOAc. The precipitate was then filtered and washed with EtOAc to afford the PEB-biguanidium **1, 2, 3** or **4** as a bis(trifluoromethane)sulfonamide salt or a trifluoromethanesulfonate salt in quantitative yields.

OTF:

MS (120.0V, ES-): *m*/*z* (%) = 148.9527 ([CF_3_O_3_SH]-H)^-^

^19^F NMR (282 MHz, DMSO-d_6_, 25 °C, TMS): δ = 77.75 (s, 3F)

NTf_2_:

MS (120.0V, ES-): *m*/*z* (%) = 279.9192 ([C_2_F_6_O_4_S_2_H]-H)^-^

^19^F NMR (282 MHz, DMSO-d_6_, 25 °C, TMS): δ = 78.73 (s, 6F)

## NMR Spectra of the synthesized compounds

^
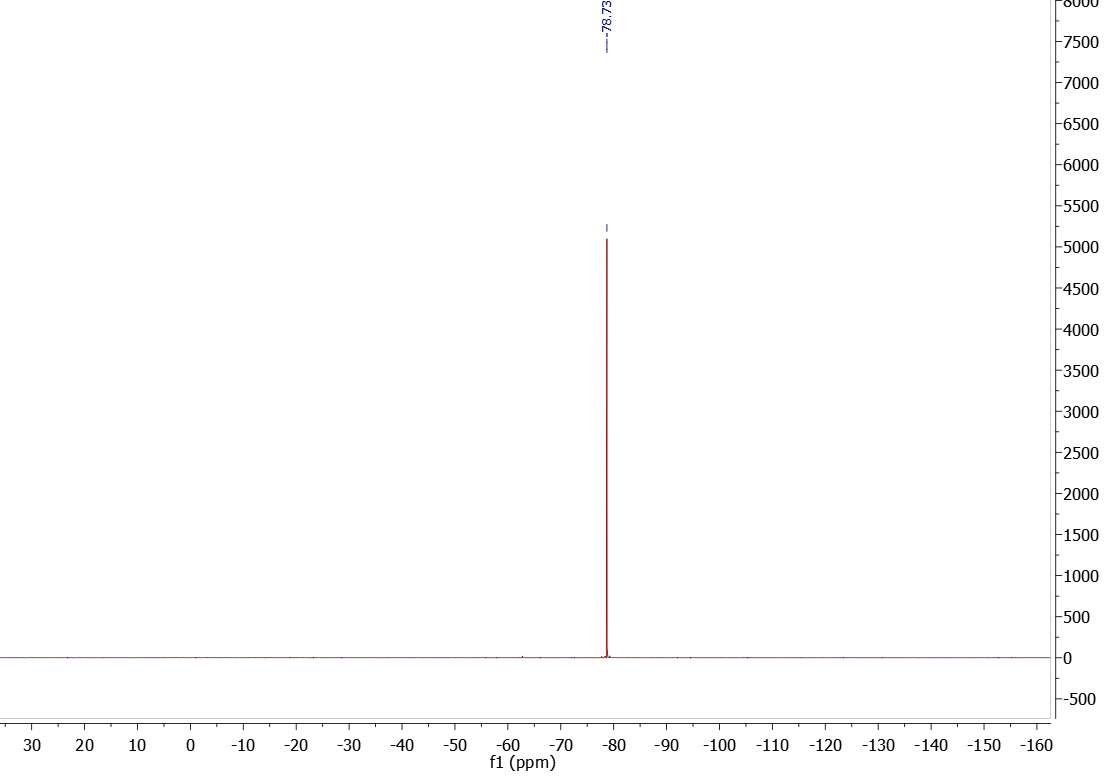
^

**Figure S1**: ^19^F NMR (282 MHz) spectrum of NTf_2_^-^ (from **1a)** in DMSO-d^6^ at 298K

^
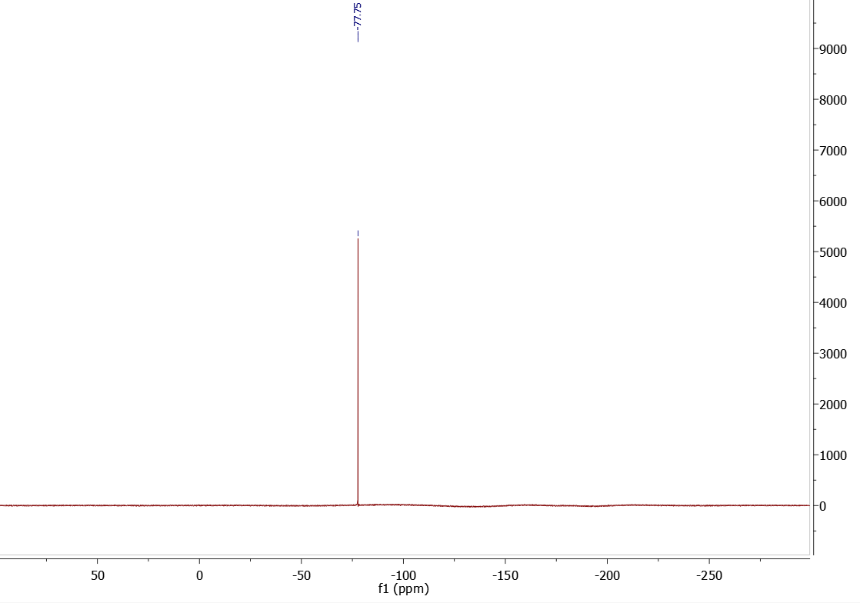
^

**Figure S2**: ^19^F NMR (282 MHz) spectrum of OTf ^-^ (from **1b**) in DMSO-d^6^ at 298K


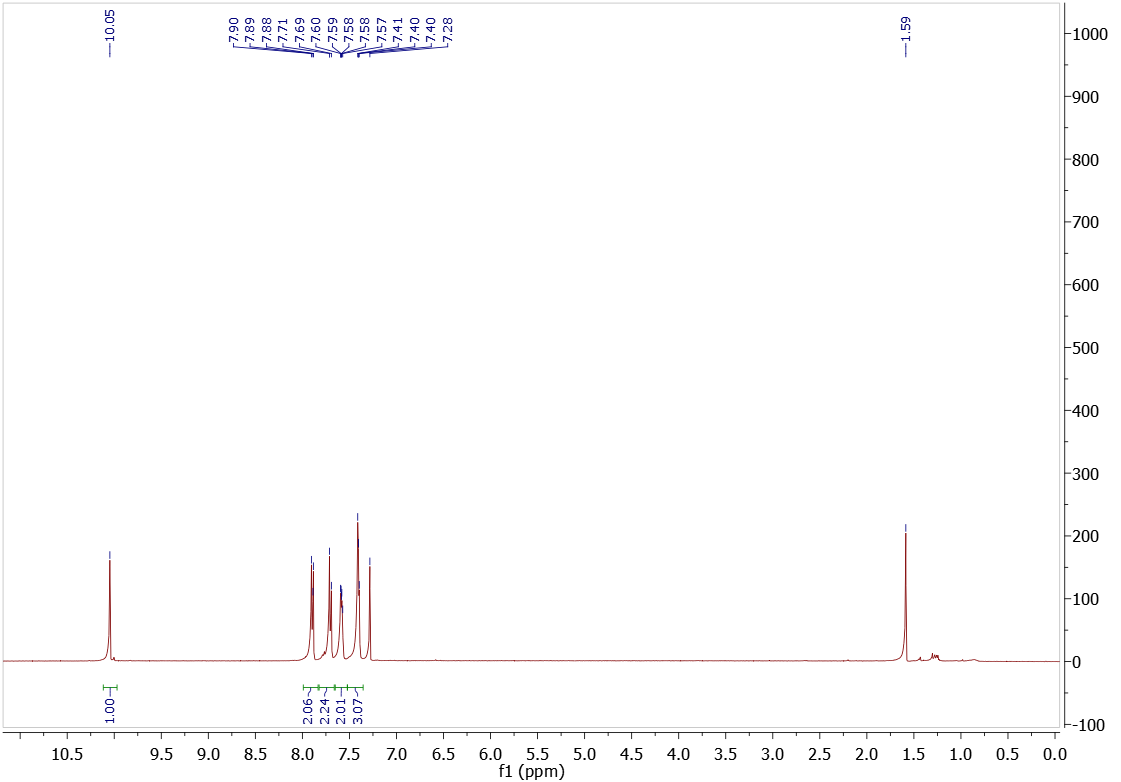


**Figure S3**: ^1^H NMR (400 MHz) spectrum of 4-(phenylethynyl)benzaldehyde in CDCl_3_ at 298K


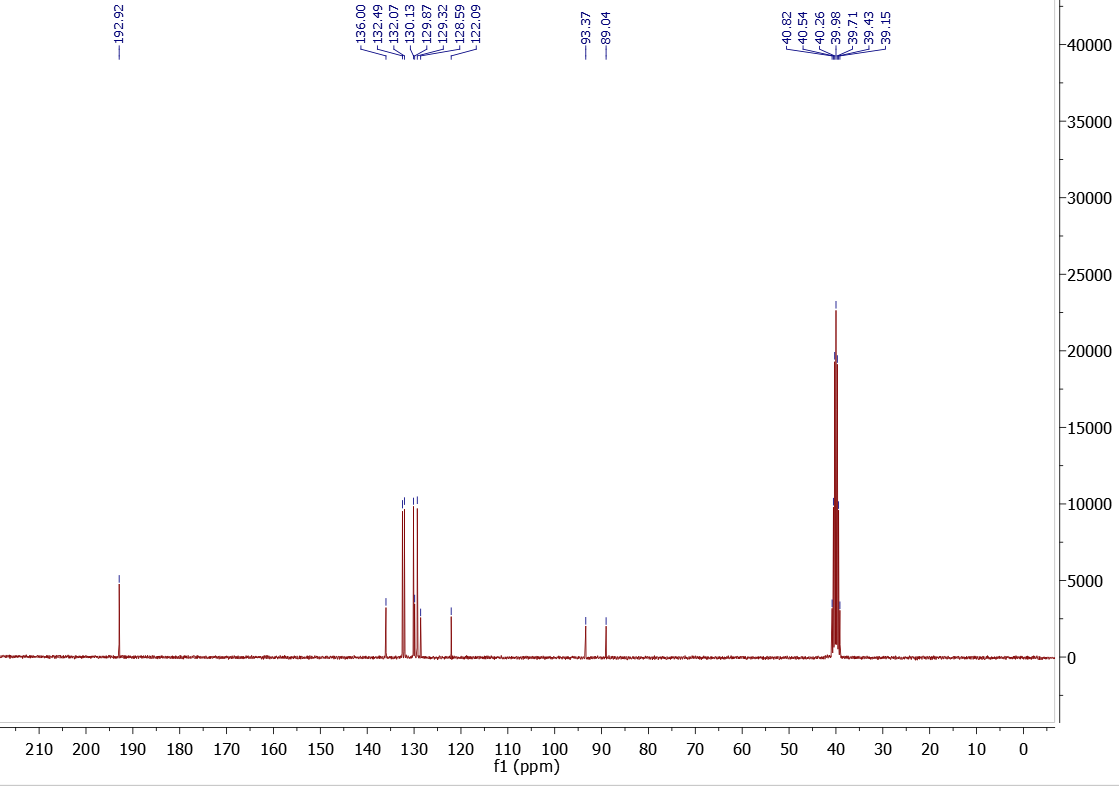


**Figure S4**: ^13^C NMR (70 MHz) spectrum of 4-(phenylethynyl)benzaldehyde in CDCl_3_ at 298K


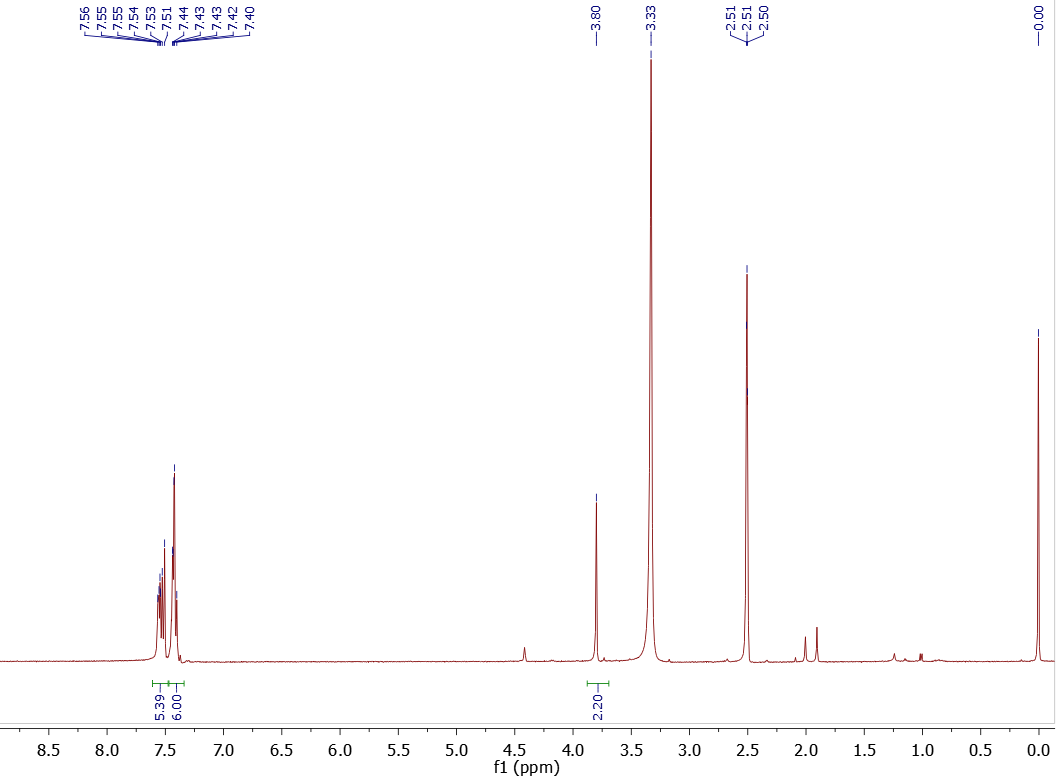


**Figure S5**: ^1^H NMR (400 MHz) spectrum of 4-(phenylethynyl)benzylamine in DMSO-d^6^ at 298K


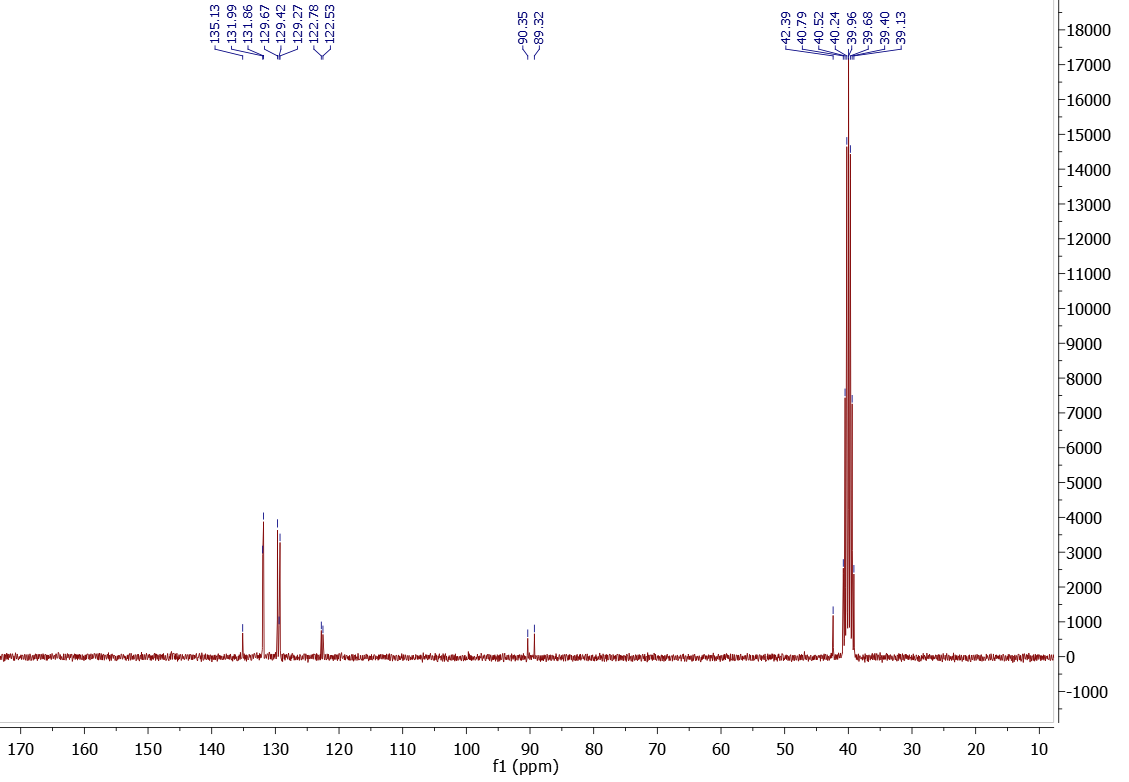


**Figure S6**: ^13^C NMR (70 MHz) spectrum of 4-(phenylethynyl)benzylamine in DMSO-d^6^ at 298K


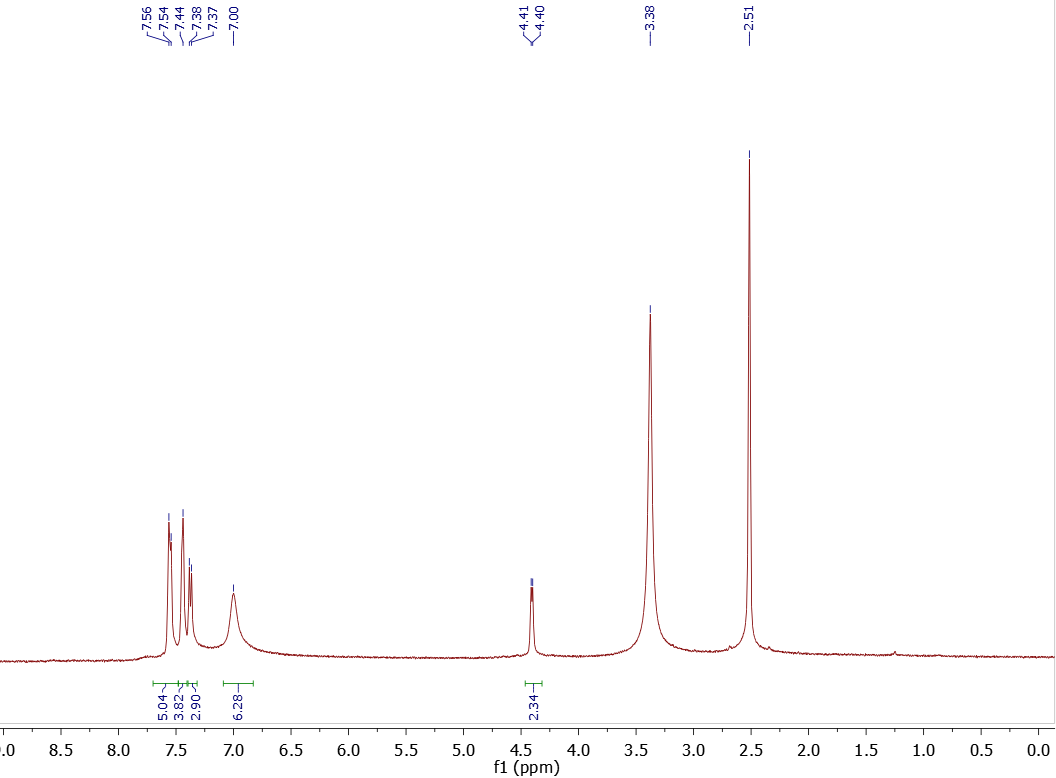


**Figure S7**: ^1^H NMR (400 MHz) spectrum of 4-(phenylethynyl)benzylbiguanide chloride salt **(1)** in DMSO-d^6^ at 298K


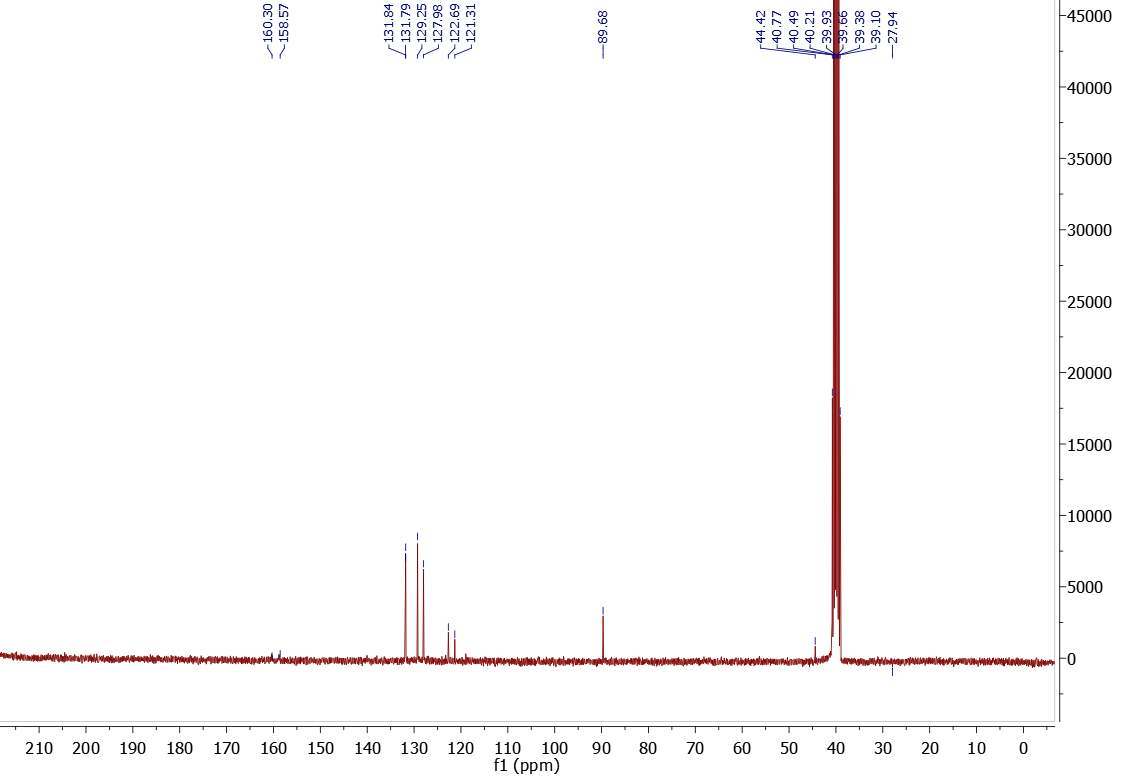


**Figure S8**: ^13^C NMR (70 MHz) spectrum of 4-(phenylethynyl)benzylbiguanide chloride salt **(1)** in DMSO-d^6^ at 298K


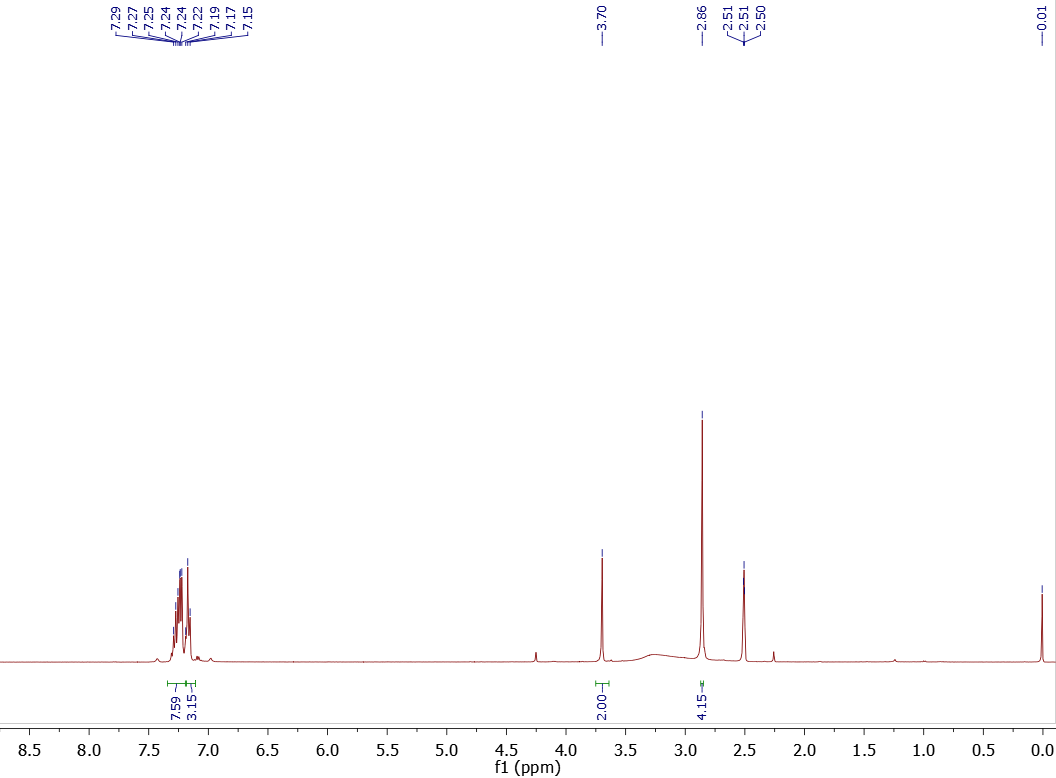


**Figure S9**: ^1^H NMR (400 MHz) spectrum of 4-(phenylethyl)benzylamine in DMSO-d^6^ at 298K


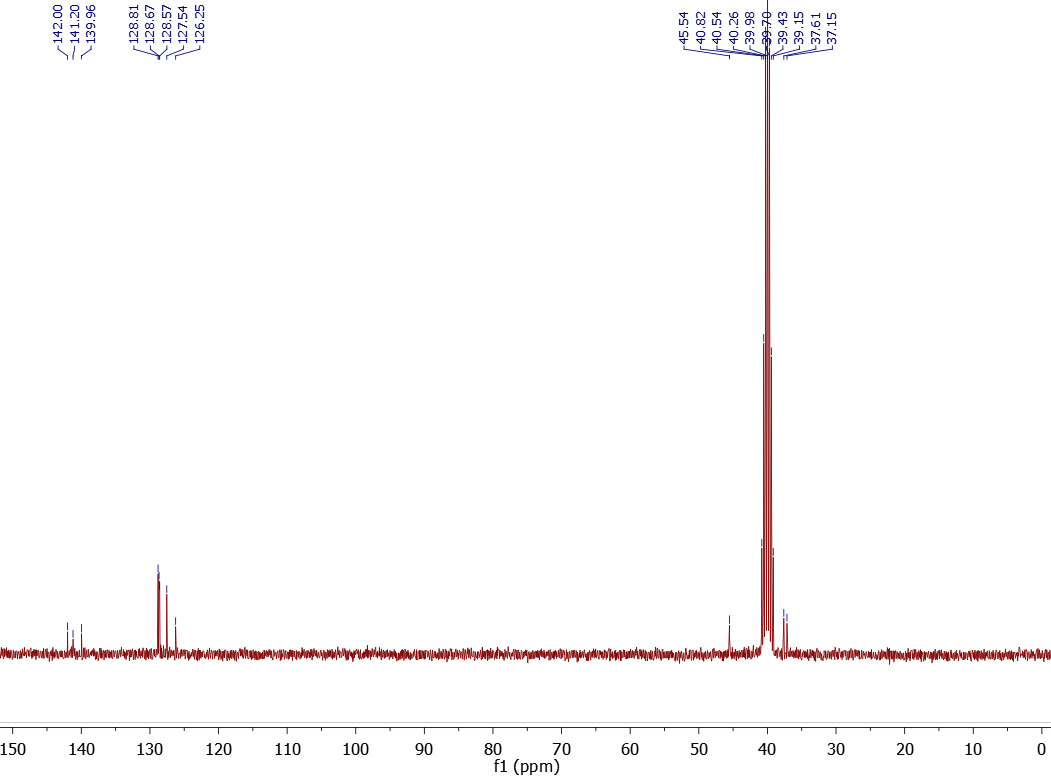


**Figure S10**: ^13^C NMR (70 MHz) spectrum of 4-(phenylethyl)benzylamine in DMSO-d^6^ at 298K


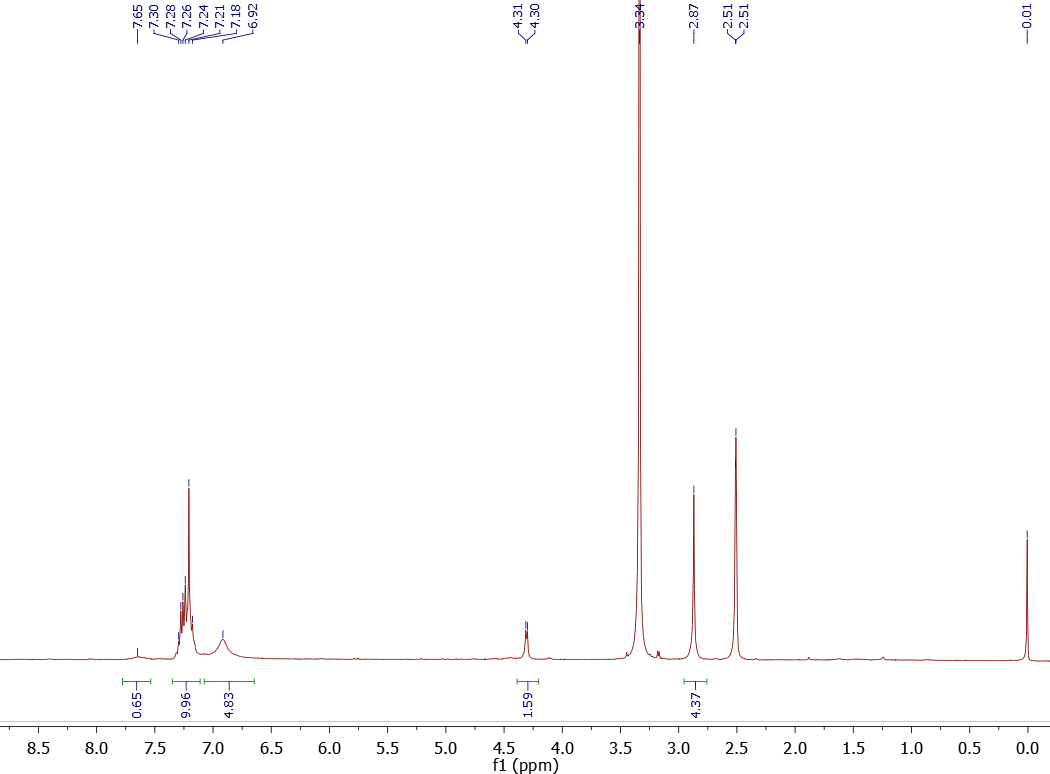


**Figure S11**: ^1^H NMR (400 MHz) spectrum of 4-(phenylethyl)benzylbiguanide chloride salt **(2)** in DMSO-d^6^ at 298K


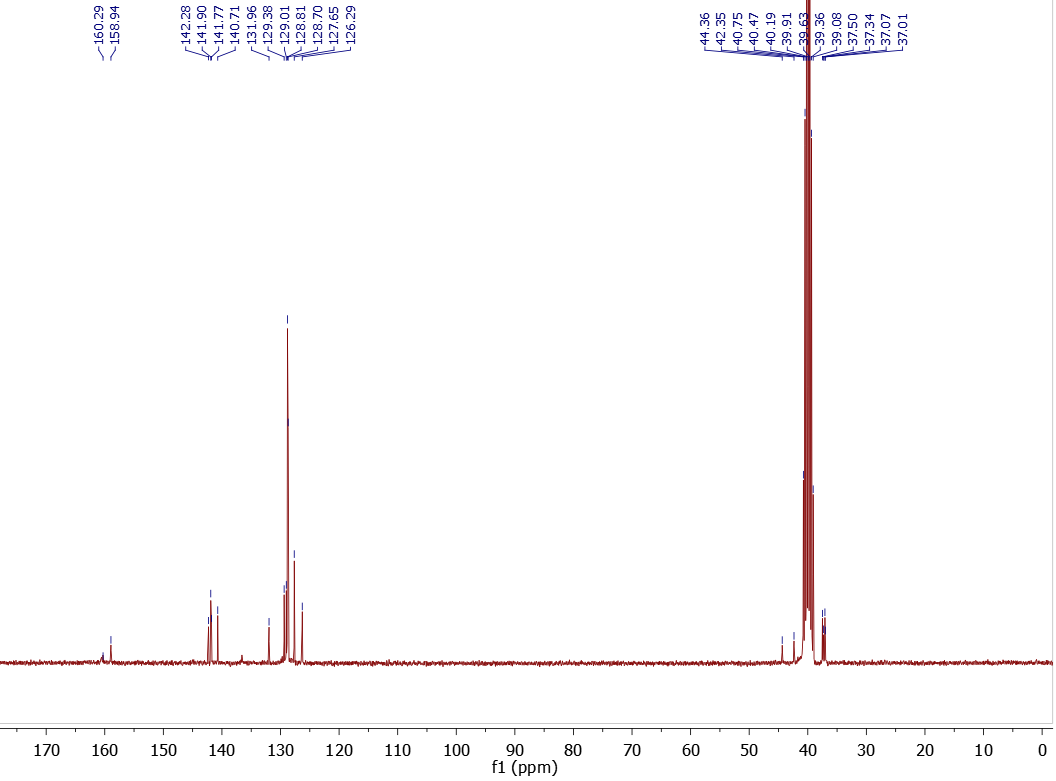


**Figure S12**: ^13^C NMR (70 MHz) spectrum of 4-(phenylethyl)benzylbiguanide chloride salt **(2)** in DMSO-d^6^ at 298K


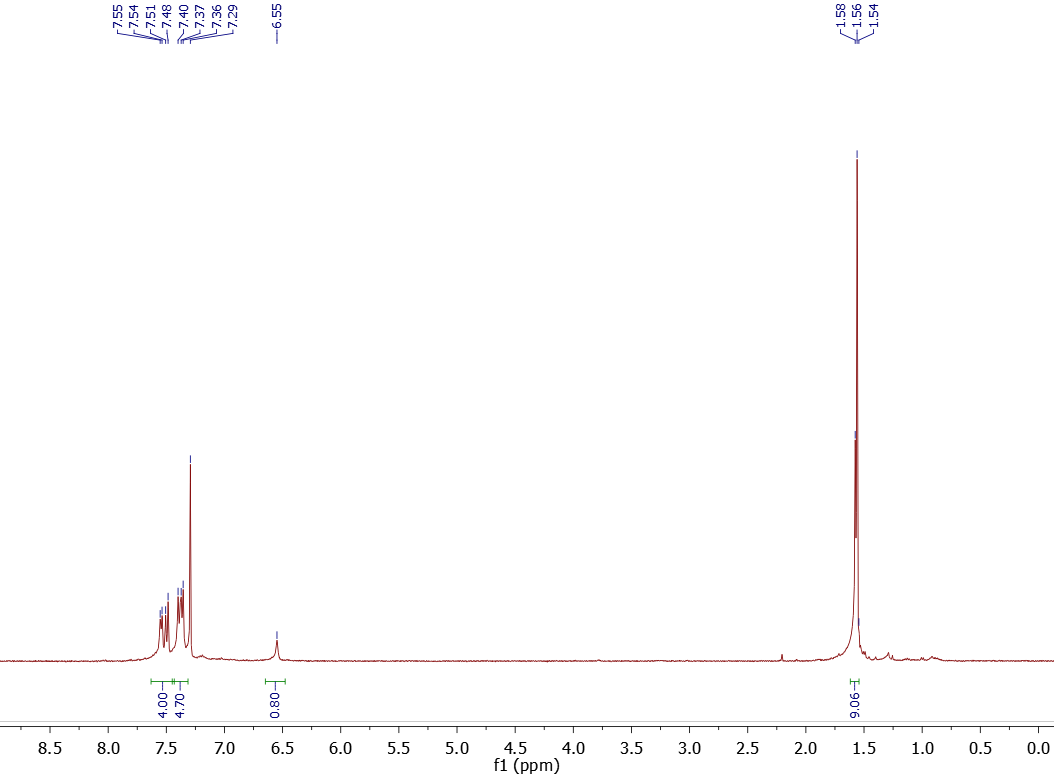


**Figure S13**: ^1^H NMR (400 MHz) spectrum of tert-butyl (4-(phenylethynyl)phenyl)carbamate in CDCl_3_ at 298K


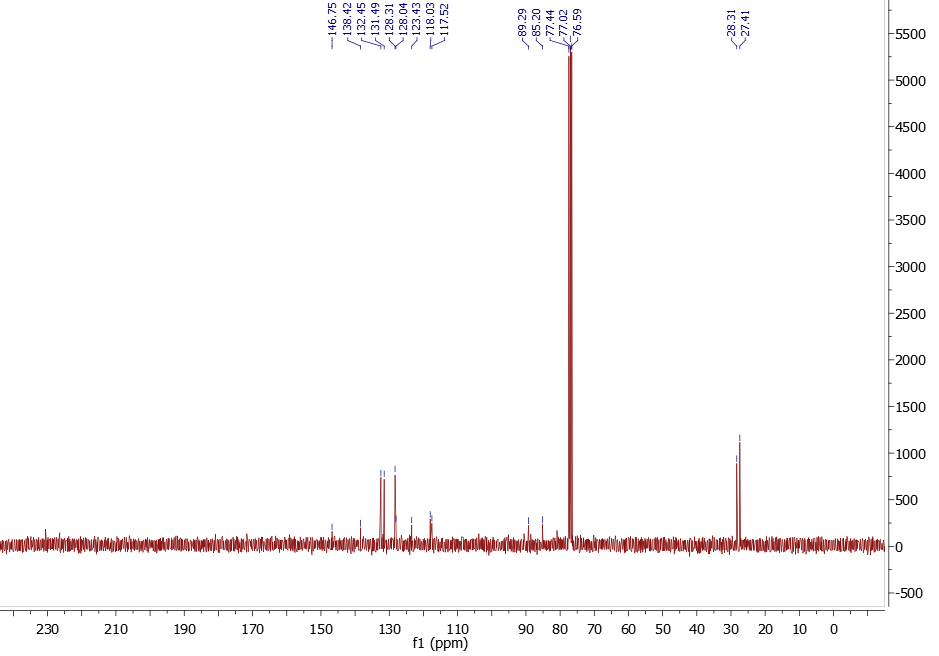


**Figure S14**: ^13^C NMR (70 MHz) spectrum of tert-butyl (4-(phenylethynyl)phenyl)carbamate in CDCl_3_ at 298K


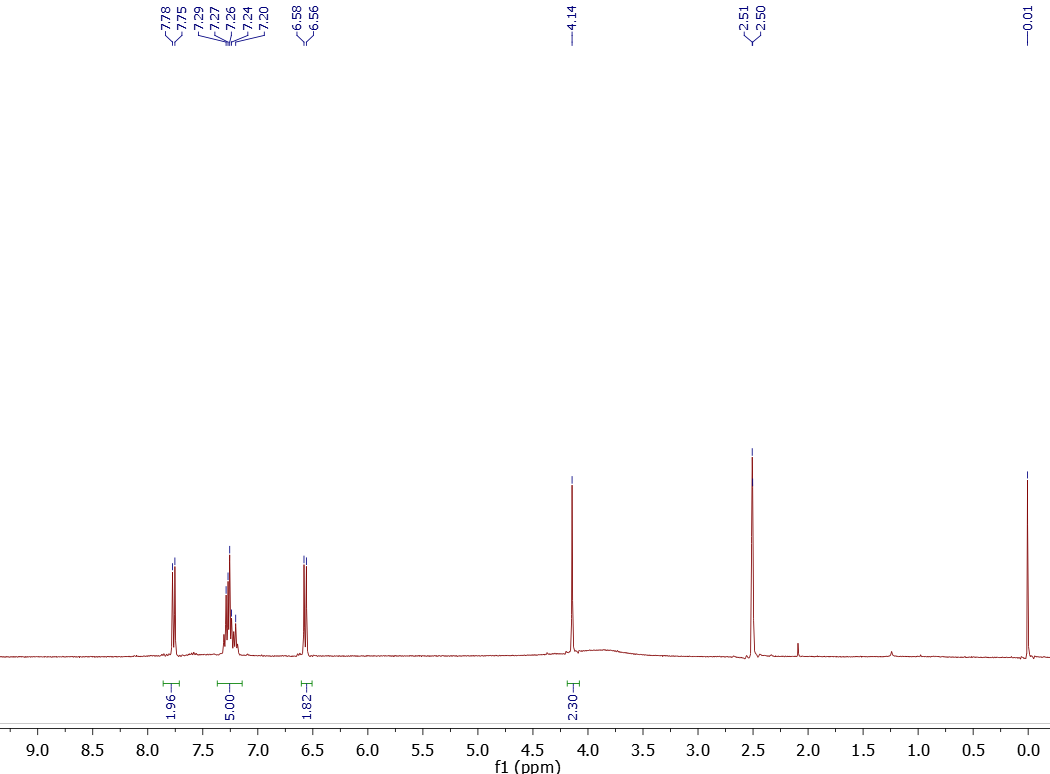


**Figure S15**: ^1^H NMR (400 MHz) spectrum of 4-(phenylethynyl)aniline trifluoroacetic salt in DMSO-d^6^ at 298K


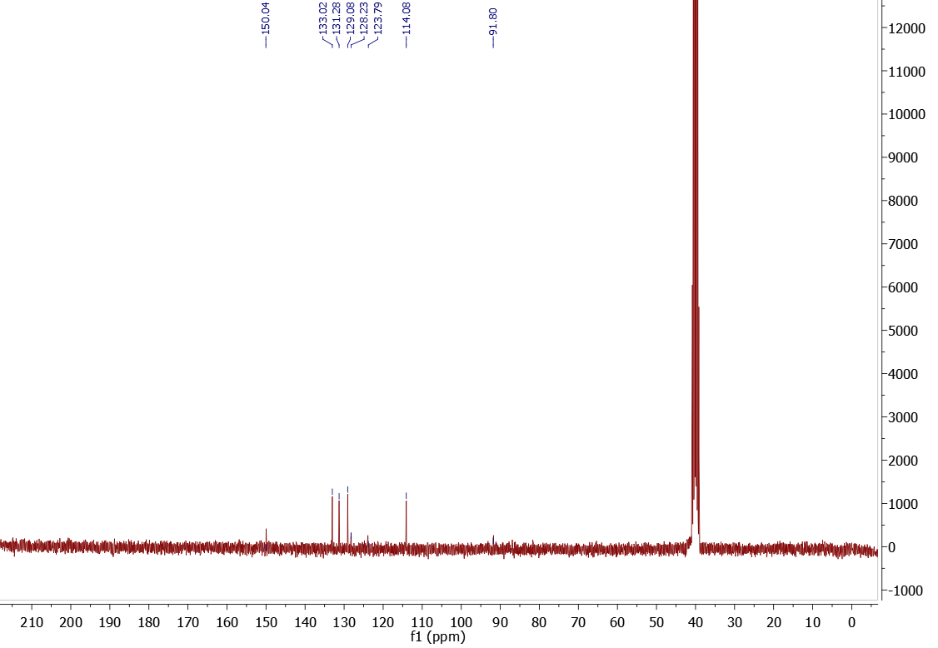


**Figure S16**: ^13^C NMR (70 MHz) spectrum of 4-(phenylethynyl)aniline trifluoroacetic salt in DMSO-d^6^ at 298K


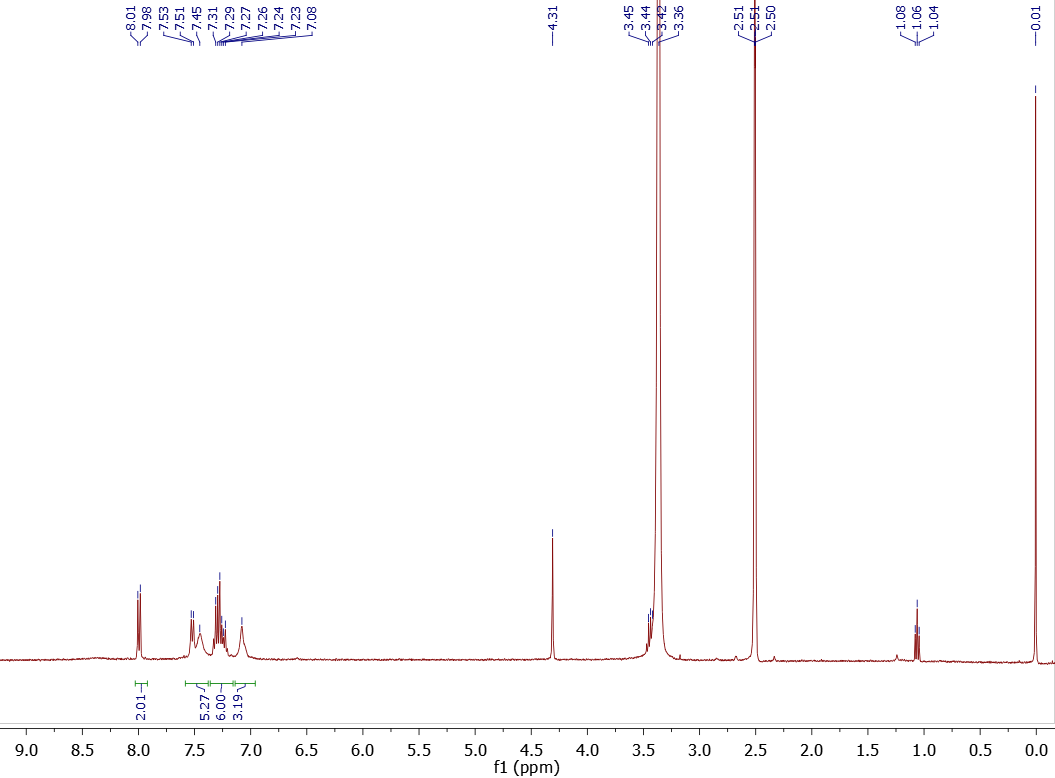


**Figure S17**: ^1^H NMR (400 MHz) spectrum of (4-(phenylethynylphenyl)biguanide trifluoroacetic salt **(3)** in DMSO-d^6^ at 298K


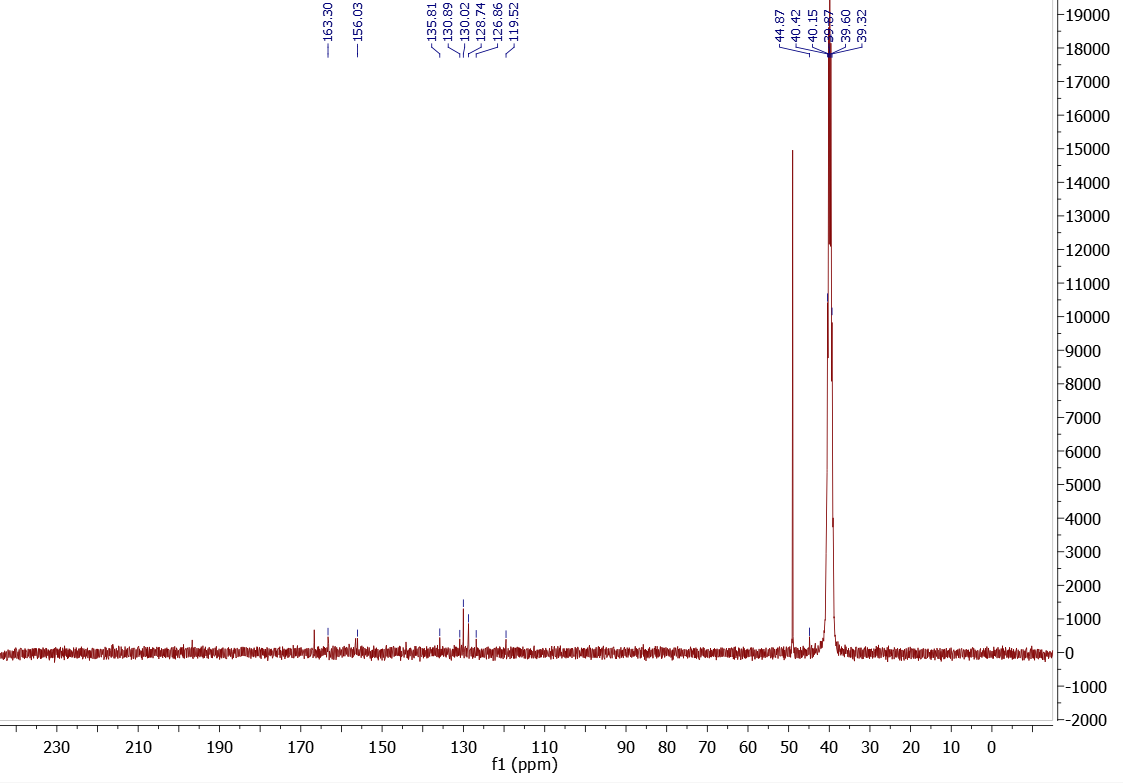


**Figure S18**: ^13^C NMR (70 MHz) spectrum of (4-(phenylethynylphenyl)biguanide trifluoroacetic salt **(3)** in DMSO-d^6^ at 298K


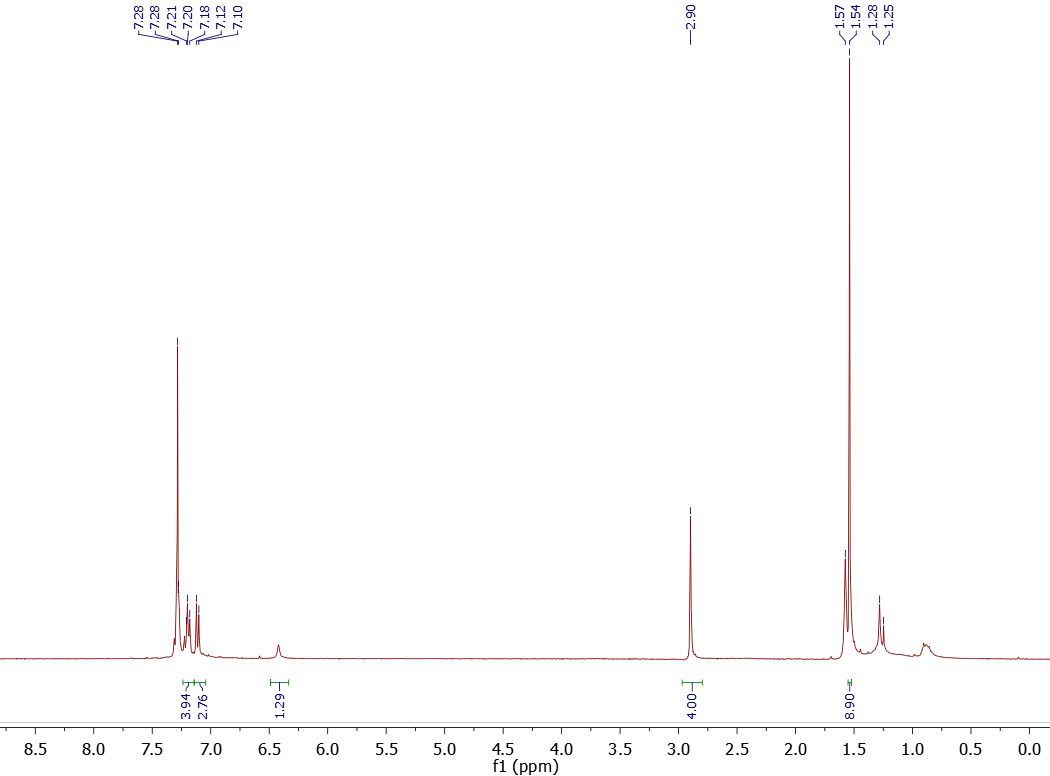


**Figure S19**: ^1^H NMR (400 MHz) spectrum of tert-butyl (4-phenethylphenyl)carbamate in CDCl_3_ at 298K


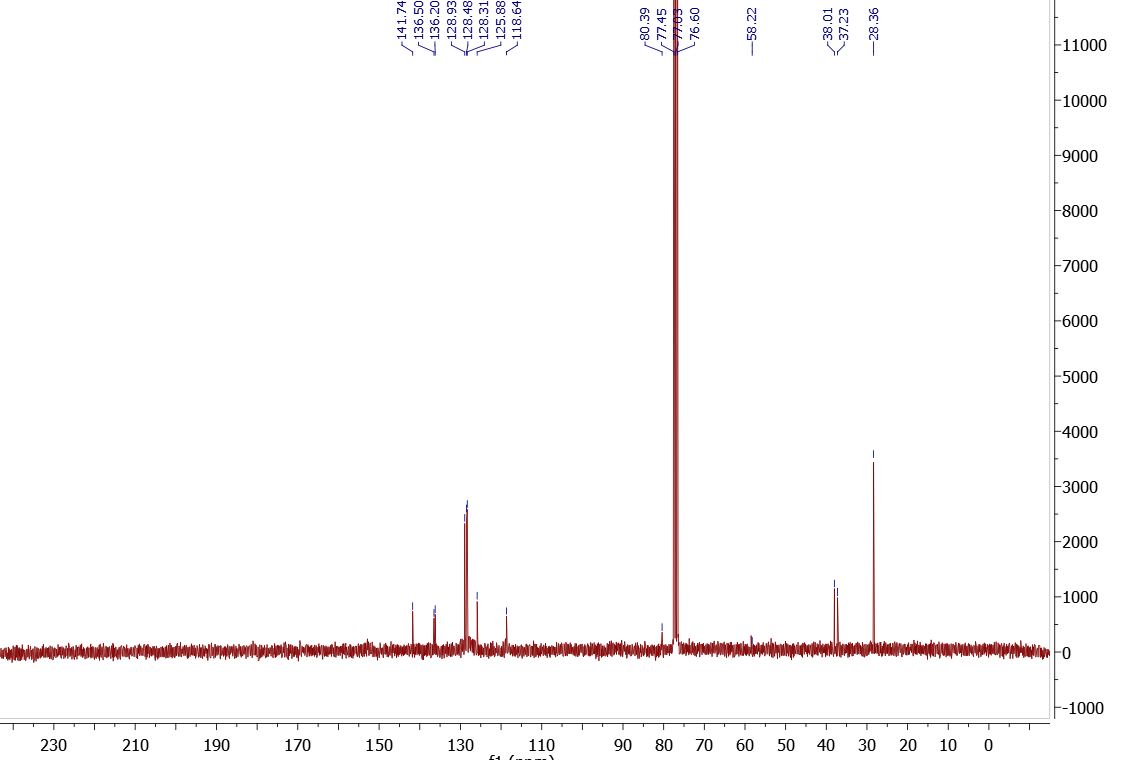


**Figure S20**: ^13^C NMR (70 MHz) spectrum of tert-butyl (4-phenethylphenyl)carbamate in CDCl_3_ at 298K


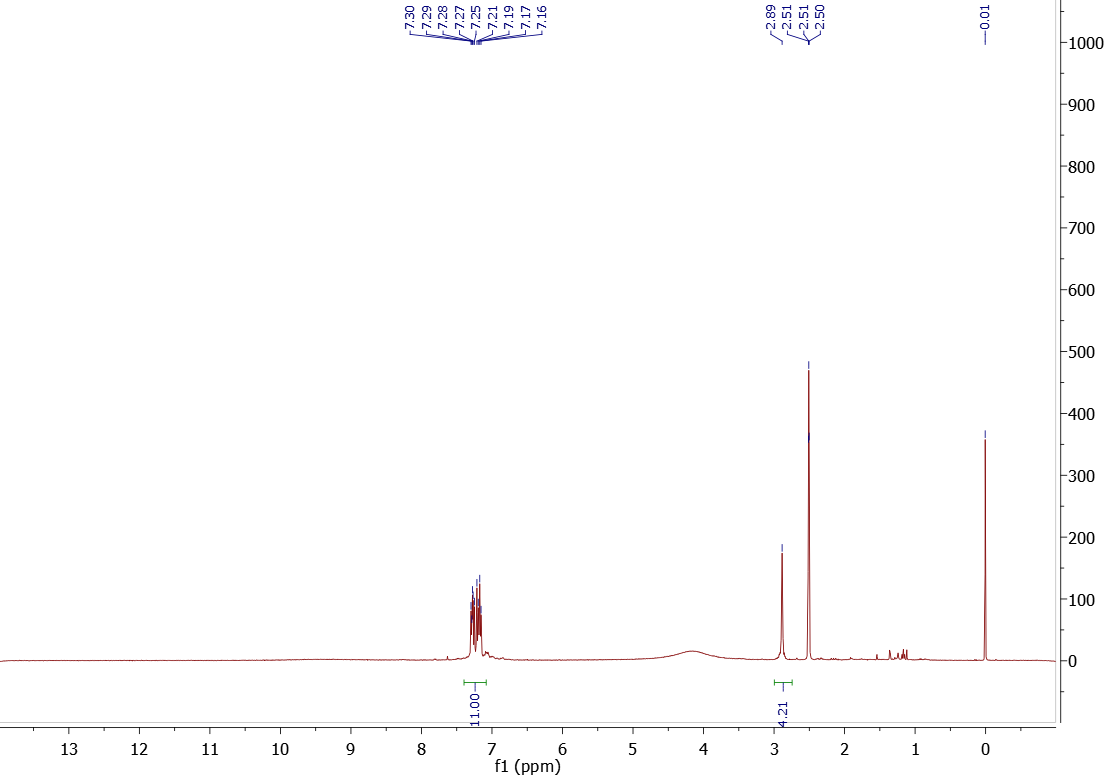


**Figure S21**: ^1^H NMR (400 MHz) spectrum of 4-(phenylethylphenyl)aniline trifluoroacetic salt in DMSO-d^6^ at 298K


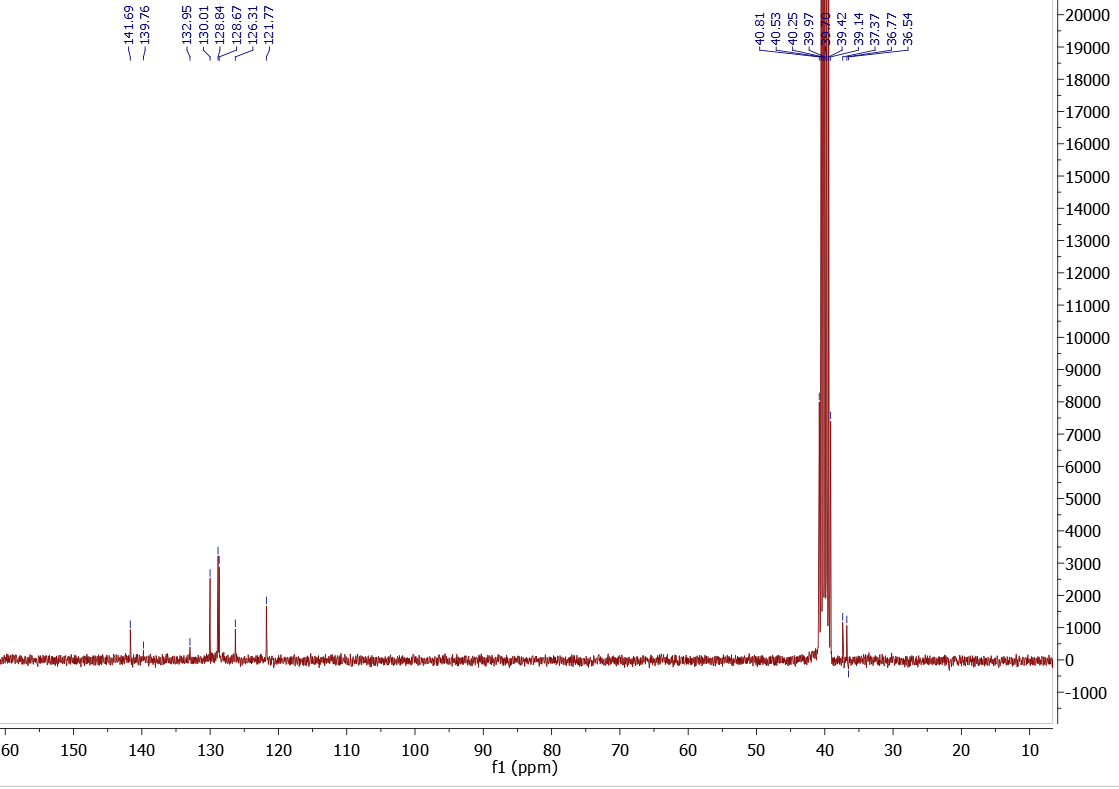


**Figure S22**: ^13^C NMR (70 MHz) spectrum of 4-(phenylethylphenyl)aniline trifluoroacetic salt in DMSO-d^6^ at 298K


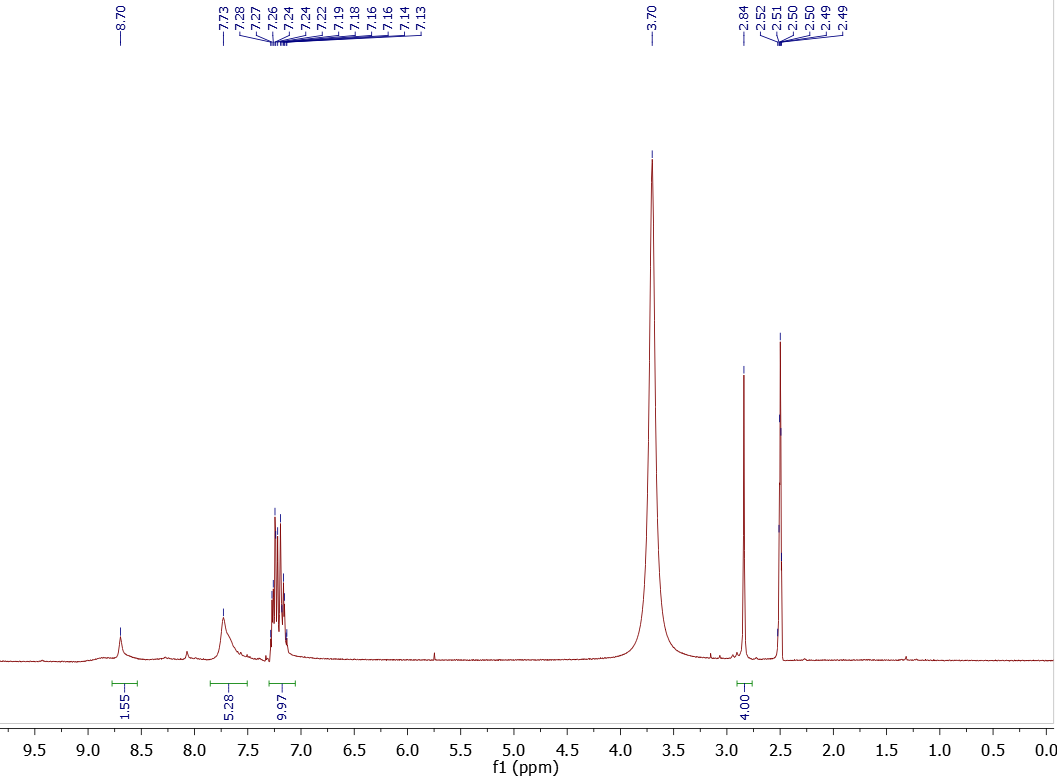


**Figure S23**: ^1^H NMR (400 MHz) spectrum of (4-(phenylethylphenyl)biguanide trifluoroacetic acid salt **(4)** in DMSO-d^6^ at 298K


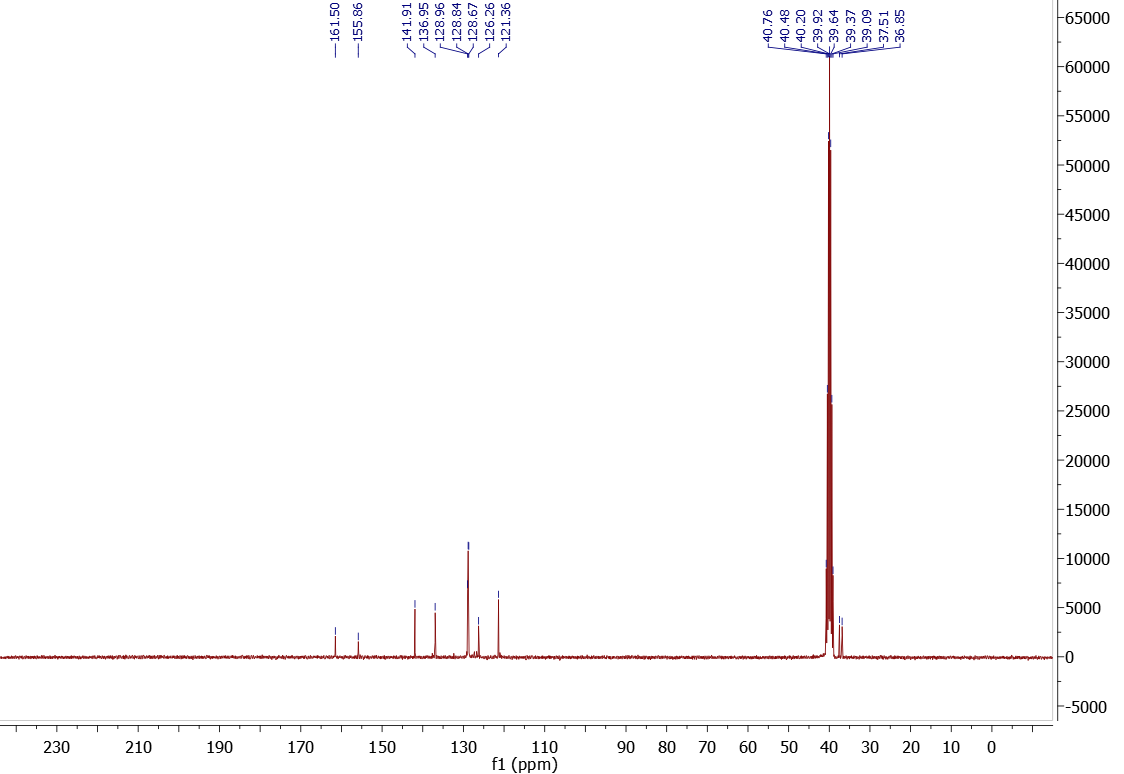


**Figure S24**: ^13^C NMR (70 MHz) spectrum of (4-(phenylethylphenyl)biguanide trifluoroacetic acid salt **(4)** in DMSO-d^6^ at 298K

# Single crystal X-ray diffraction

**Experimental.** Single crystals of C_18_H_18_F_3_N_5_O_3_S were obtained from chlorofom. A suitable crystal was selected and analysed on a **Bruker Venture Metaljet** diffractometer. The crystal was kept at 100 K during data collection. Using Olex, the structure was solved with the XT structure solution program using Intrinsic Phasing and refined with the XL refinement package using Least Squares minimisation.

**Crystal structure determination of 1b**

**Crystal Data** for C_18_H_18_F_3_N_5_O_3_S (*M*=441.43 g/mol): orthorhombic, space group Pca2_1_ (no. 29), *a* = 15.3543(10) Å, *b* = 5.8490(4) Å, *c* = 43.017(3) Å, *V*= 3863.2(4) Å^3^, *Z* = 8, *T* = 100 K, μ(GaKα) = 1.321 mm^-1^, *Dcalc* = 1.518 g/cm^3^, 81637 reflections measured (7.152° ≤ 2Θ ≤ 126.968°), 9587 unique (*R*_int_ = 0.0481, R_sigma_ = 0.0246) which were used in all calculations. The final *R*_1_ was 0.0361 (I > 2σ(I)) and *wR*_2_ was 0.0952 (all data).

**Refinement model description**

Number of restraints - 1, number of constraints - unknown. Details:

1. Twinned data refinement
 Scales: 0.78(2) 0.22(2)
2. Fixed Uiso
 At 1.2 times of:
 All C(H) groups, All C(H,H) groups
3.a Secondary CH2 refined with riding coordinates:
 C3(H3A,H3B), C21(H21A,H21B)
3.b Aromatic/amide H refined with riding coordinates:
 C5(H5A), C6(H6), C8(H8), C9(H9), C13(H13), C14(H14), C15(H15), C16(H16),
 C17(H17), C23(H23), C24(H24), C26(H26), C27(H27), C31(H31), C32(H32), C33(H33),
 C34(H34), C35(H35)

**Figure S25**: Crystal packing of compound **1b**

| **Table S1.**Crystal data and structure refinement for compound **1b** | |
| --- | --- |
| Identification code | 1b |
| Empirical formula | C_18_H_18_F_3_N_5_O_3_S |
| Formula weight | 441.43 |
| Temperature/K | 100 |
| Crystal system | orthorhombic |
| Space group | Pca2_1_ |
| a/Å | 15.3543(10) |
| b/Å | 5.8490(4) |
| c/Å | 43.017(3) |
| α/° | 90 |
| β/° | 90 |
| γ/° | 90 |
| Volume/Å^3^ | 3863.2(4) |
| Z | 8 |
| ρ_calc_g/cm^3^ | 1.518 |
| μ/mm^‑1^ | 1.321 |
| F(000) | 1824.0 |
| Crystal size/mm^3^ | 0.34 × 0.14 × 0.11 |
| Radiation | GaKα (λ = 1.34139) |
| 2Θ range for data collection/° | 7.152 to 126.968 |
| Index ranges | -17 ≤ h ≤ 20, -7 ≤ k ≤ 7, -57 ≤ l ≤ 57 |
| Reflections collected | 81637 |
| Independent reflections | 9587 [R_int_ = 0.0481, R_sigma_ = 0.0246] |
| Data/restraints/parameters | 9587/1/599 |
| Goodness-of-fit on F^2^ | 1.059 |
| Final R indexes [I>=2σ (I)] | R_1_ = 0.0361, wR_2_ = 0.0943 |
| Final R indexes [all data] | R_1_ = 0.0372, wR_2_ = 0.0952 |
| Largest diff. peak/hole / e Å^-3^ | 0.34/-0.61 |
| Flack parameter | 0.22(2) |

| **Table S2.** Fractional Atomic Coordinates (×10^4^) and Equivalent Isotropic Displacement Parameters (Å^2^×10^3^) for compound 1b. U_eq_ is defined as 1/3 of of the trace of the orthogonalised U_IJ_ tensor. | | | | |
| --- | --- | --- | --- | --- |
| **Atom** | ***x*** | ***y*** | ***z*** | **U(eq)** |
| S1 | 4827.7(4) | 3450.5(10) | 4584.9(2) | 14.40(13) |
| F1 | 5669.4(11) | 30(3) | 4836.1(5) | 28.3(4) |
| F2 | 4285.2(11) | -12(3) | 4915.9(4) | 24.6(4) |
| F3 | 4793.7(13) | -931(3) | 4465.6(4) | 26.7(4) |
| O1 | 5538.7(14) | 3679(4) | 4364.1(5) | 24.8(4) |
| O2 | 4920.9(14) | 4715(4) | 4869.3(6) | 25.4(5) |
| O3 | 3969.4(14) | 3569(3) | 4443.9(5) | 21.5(4) |
| C18 | 4904.1(17) | 464(5) | 4706.7(6) | 16.3(5) |
| S2 | 2468.6(4) | 11553.4(10) | 5398.3(2) | 14.62(13) |
| F4 | 1629.0(11) | 14971(3) | 5146.9(5) | 29.1(4) |
| F5 | 3011.6(11) | 15008(3) | 5066.3(4) | 25.6(4) |
| F6 | 2504.1(12) | 15936(3) | 5517.7(4) | 26.9(4) |
| O4 | 2375.5(14) | 10287(4) | 5114.7(5) | 25.7(5) |
| O5 | 3329.1(13) | 11434(3) | 5540.4(5) | 21.6(4) |
| O6 | 1762.5(14) | 11322(4) | 5617.7(5) | 24.3(4) |
| C36 | 2393.3(17) | 14538(5) | 5278.3(6) | 16.7(5) |
| N1 | 1171.2(17) | 12626(5) | 3977.1(6) | 22.8(5) |
| N2 | 2043.4(16) | 12865(4) | 4407.7(5) | 18.6(4) |
| N3 | 2239.3(14) | 9955(4) | 4026.8(5) | 16.3(4) |
| N4 | 2664.3(17) | 8290(4) | 4510.6(5) | 17.8(5) |
| N5 | 3216.6(15) | 7013(4) | 4051.8(5) | 16.0(4) |
| C1 | 1824.2(17) | 11771(5) | 4144.3(6) | 15.5(5) |
| C2 | 2699.6(18) | 8511(4) | 4200.4(6) | 14.6(5) |
| C3 | 3301.7(17) | 6869(5) | 3715.1(6) | 15.6(5) |
| C4 | 4075.3(16) | 8180(4) | 3587.5(6) | 14.0(5) |
| C5 | 4328.5(17) | 10272(5) | 3714.3(6) | 16.7(5) |
| C6 | 5006.0(19) | 11517(5) | 3583.0(7) | 18.0(5) |
| C7 | 5437.2(17) | 10689(5) | 3316.7(6) | 16.9(5) |
| C8 | 5186.7(17) | 8563(5) | 3192.9(6) | 17.8(5) |
| C9 | 4516.8(17) | 7330(5) | 3327.6(6) | 15.8(5) |
| C10 | 6129.6(18) | 11993(5) | 3179.3(7) | 19.2(5) |
| C11 | 6698.0(19) | 13128(5) | 3072.8(7) | 19.8(5) |
| C12 | 7387.1(17) | 14580(5) | 2961.1(6) | 17.1(5) |
| C13 | 7513.9(18) | 16731(5) | 3099.3(7) | 19.4(5) |
| C14 | 8178(2) | 18147(5) | 2995.8(7) | 22.6(6) |
| C15 | 8717.3(19) | 17456(6) | 2752.5(7) | 24.2(6) |
| C16 | 8599(2) | 15318(6) | 2615.8(7) | 23.9(6) |
| C17 | 7938.3(19) | 13894(5) | 2717.7(6) | 21.0(5) |
| N6 | 6127.4(17) | 2377(5) | 6005.8(6) | 22.5(5) |
| N7 | 5259.0(16) | 2150(4) | 5576.5(6) | 18.0(4) |
| N8 | 5060.3(14) | 5060(4) | 5957.6(6) | 17.2(4) |
| N9 | 4634.4(17) | 6723(4) | 5473.2(5) | 19.0(5) |
| N10 | 4087.6(15) | 8006(4) | 5933.1(5) | 15.9(4) |
| C19 | 5470.0(17) | 3242(5) | 5838.9(6) | 14.9(5) |
| C20 | 4603.1(17) | 6512(4) | 5785.2(6) | 14.3(5) |
| C21 | 4000.9(17) | 8144(4) | 6267.3(6) | 15.6(5) |
| C22 | 3226.8(17) | 6830(4) | 6393.7(6) | 14.4(5) |
| C23 | 2971.7(17) | 4735(5) | 6265.8(6) | 17.0(5) |
| C24 | 2301.0(19) | 3480(5) | 6396.6(6) | 18.0(5) |
| C25 | 1859.2(17) | 4292(5) | 6660.0(6) | 16.9(5) |
| C26 | 2099.5(18) | 6414(5) | 6786.2(6) | 16.7(5) |
| C27 | 2774.9(17) | 7666(5) | 6652.7(6) | 15.5(5) |
| C28 | 1174.0(18) | 2953(5) | 6795.6(6) | 18.5(5) |
| C29 | 608.4(19) | 1764(5) | 6902.1(6) | 18.1(5) |
| C30 | -56.4(17) | 281(5) | 7017.5(7) | 17.1(5) |
| C31 | -203.5(18) | -1842(5) | 6874.0(7) | 18.4(5) |
| C32 | -846(2) | -3285(5) | 6984.6(7) | 22.1(6) |
| C33 | -1354.7(19) | -2639(6) | 7237.8(7) | 25.8(6) |
| C34 | -1219.7(19) | -556(6) | 7380.5(7) | 23.9(6) |
| C35 | -579.4(19) | 912(5) | 7273.4(7) | 20.3(5) |

| **Table S3.** Anisotropic Displacement Parameters (Å^2^×10^3^) for compound 1b. The Anisotropic displacement factor exponent takes the form: -2π^2^[h^2^a*^2^U_11_+2hka*b*U_12_+…]. | | | | | | |
| --- | --- | --- | --- | --- | --- | --- |
| **Atom** | **U_11_** | **U_22_** | **U_33_** | **U_23_** | **U_13_** | **U_12_** |
| S1 | 11.0(3) | 15.5(3) | 16.7(3) | 0.5(2) | -0.9(2) | 0.2(2) |
| F1 | 16.9(8) | 28.8(9) | 39.1(10) | 7.7(8) | -7.3(7) | 7.9(8) |
| F2 | 22.2(8) | 28.5(9) | 23.1(8) | 10.1(7) | 7.2(6) | 2.3(8) |
| F3 | 36.8(10) | 17.5(8) | 25.9(9) | -3.2(7) | 1.1(7) | -0.5(7) |
| O1 | 20.2(10) | 23.5(10) | 30.6(11) | 4.6(8) | 8.2(8) | -2.9(8) |
| O2 | 27.7(11) | 24.1(10) | 24.5(12) | -6.5(9) | -3.5(8) | 1.5(9) |
| O3 | 16.6(9) | 20.7(10) | 27.1(10) | 6.8(8) | -6.8(8) | -0.1(8) |
| C18 | 12.3(11) | 18.8(12) | 17.9(12) | 2.5(10) | 0.4(9) | 0.8(9) |
| S2 | 11.3(3) | 16.1(3) | 16.5(3) | 0.6(2) | -1.0(2) | -0.1(2) |
| F4 | 16.8(7) | 30.2(9) | 40.2(11) | 6.9(8) | -6.8(7) | 6.2(8) |
| F5 | 22.2(8) | 29.6(9) | 24.9(8) | 10.3(7) | 7.7(7) | 0.8(8) |
| F6 | 37.5(11) | 18.5(8) | 24.6(9) | -2.7(7) | 0.2(7) | -0.2(7) |
| O4 | 29.1(11) | 23.9(11) | 24.1(11) | -7.4(9) | -3.6(8) | 1.8(9) |
| O5 | 16.1(9) | 23.1(10) | 25.5(10) | 6.5(8) | -7.7(8) | -0.5(7) |
| O6 | 20.6(10) | 24.1(10) | 28.1(10) | 3.6(8) | 8.6(8) | -2.8(8) |
| C36 | 14.5(11) | 20.4(13) | 15.3(12) | 2.4(10) | 0.3(9) | 0.7(9) |
| N1 | 18.2(11) | 25.8(12) | 24.4(12) | -7.6(10) | -5.4(9) | 8.5(10) |
| N2 | 16.0(11) | 19.3(11) | 20.4(11) | -4.6(9) | -1.4(8) | 2.5(9) |
| N3 | 15.7(10) | 18.2(11) | 15.1(10) | -0.7(8) | 0.3(8) | 1.8(10) |
| N4 | 18.8(11) | 19.6(11) | 15.0(11) | 2.3(9) | -0.4(9) | 2.6(10) |
| N5 | 14.3(10) | 17.7(10) | 16.2(10) | 2.8(8) | 0.4(8) | 3.1(8) |
| C1 | 11.1(11) | 16.2(11) | 19.2(12) | 1.6(9) | 2.0(9) | -1.5(9) |
| C2 | 11.2(11) | 15.5(12) | 17.1(11) | 0.5(9) | 0.2(9) | -3.4(9) |
| C3 | 14.4(11) | 16.6(11) | 15.7(11) | -1.9(9) | 1.4(9) | -0.9(9) |
| C4 | 11.9(11) | 15.6(11) | 14.4(11) | 1.4(9) | -1.3(8) | 0.8(9) |
| C5 | 17.8(12) | 17.7(12) | 14.8(11) | -0.1(9) | -0.6(9) | -2.2(10) |
| C6 | 17.8(12) | 16.2(12) | 20.1(12) | 0.6(9) | -2.7(10) | -1.2(10) |
| C7 | 13.2(11) | 19.3(12) | 18.1(12) | 3.1(10) | -1.6(9) | -0.9(9) |
| C8 | 13.6(12) | 22.1(13) | 17.5(11) | -1(1) | 0.7(9) | 3(1) |
| C9 | 15.1(11) | 16.9(12) | 15.4(11) | -1.6(9) | -2.1(9) | 2.3(9) |
| C10 | 16.7(13) | 20.1(12) | 20.8(12) | 1(1) | -1.6(10) | -0.3(10) |
| C11 | 18.7(13) | 22.7(13) | 18.1(11) | 2.3(10) | 0.1(10) | -0.4(10) |
| C12 | 14.4(11) | 20.6(12) | 16.4(12) | 4.2(10) | -3.3(9) | -3.4(10) |
| C13 | 16.5(13) | 21.0(14) | 20.7(12) | 2.1(11) | -2.3(10) | 1(1) |
| C14 | 23.8(14) | 20.0(12) | 24.0(13) | 2.2(10) | -6.3(10) | -4.3(11) |
| C15 | 21.3(13) | 30.1(15) | 21.3(13) | 5.4(11) | -1.8(11) | -9.7(12) |
| C16 | 21.3(13) | 33.5(15) | 16.9(12) | 3.3(11) | 0.6(10) | -4.1(12) |
| C17 | 23.4(13) | 23.0(13) | 16.7(11) | 2.1(10) | -1.3(10) | -3.5(11) |
| N6 | 19.5(11) | 25.2(12) | 22.9(12) | -6.8(10) | -4.4(9) | 8.4(10) |
| N7 | 13.4(11) | 19.0(11) | 21.7(11) | -2.7(9) | -2.6(9) | 1.7(9) |
| N8 | 15.1(10) | 21.0(12) | 15.5(11) | 0.3(8) | 0.1(7) | 2.8(10) |
| N9 | 18.8(11) | 22.2(12) | 16.1(11) | 2.1(9) | 0.7(8) | 1.2(10) |
| N10 | 14.7(10) | 17.9(10) | 15(1) | 3.7(8) | -1.5(8) | 1.7(9) |
| C19 | 12.0(11) | 18.6(12) | 13.9(11) | 1.2(9) | 3.0(9) | -2.1(9) |
| C20 | 11.0(11) | 16.6(12) | 15.2(11) | 0.1(9) | 1.3(9) | -3.8(9) |
| C21 | 13.0(11) | 16.9(11) | 17.0(11) | -1.1(9) | 1.4(9) | -1.6(9) |
| C22 | 12.5(11) | 15.5(11) | 15.1(11) | 1.8(9) | -2.1(9) | 1.1(9) |
| C23 | 16.0(12) | 19.0(12) | 16.0(11) | -1.2(9) | 0.5(9) | 1.1(10) |
| C24 | 18.6(12) | 17.1(13) | 18.3(12) | -1.2(9) | -1.7(10) | -2.3(9) |
| C25 | 13.9(11) | 20.6(12) | 16.4(11) | 4.5(9) | -1.6(9) | -2.7(9) |
| C26 | 14.9(12) | 20.6(13) | 14.6(11) | 0.0(9) | 2.0(9) | 0.1(10) |
| C27 | 13.9(11) | 16.6(11) | 16.0(11) | -0.7(9) | -2.0(9) | -0.7(9) |
| C28 | 17.1(12) | 21.2(13) | 17.1(12) | 1.9(10) | -0.4(10) | -2.6(10) |
| C29 | 16.1(12) | 20.4(13) | 17.8(11) | 0.3(9) | -2.8(10) | -1.6(10) |
| C30 | 14.2(11) | 18.9(12) | 18.2(12) | 2.1(10) | -3.0(9) | 0(1) |
| C31 | 17.7(12) | 19.8(13) | 17.7(12) | -0.4(10) | -1.5(9) | -1.1(10) |
| C32 | 22.7(14) | 21.1(13) | 22.5(13) | 1.7(10) | -6.1(11) | -5.2(11) |
| C33 | 18.4(13) | 33.8(15) | 25.3(14) | 10.4(12) | -1.4(11) | -8.6(12) |
| C34 | 17.9(12) | 34.2(15) | 19.5(12) | 4.5(12) | 1.8(10) | 2.3(11) |
| C35 | 18.8(12) | 22.3(13) | 19.8(12) | -0.7(10) | -4.3(10) | 0.7(11) |

| **Table S4.** Bond Lengths for compound **1b.** | | | | | | |
| --- | --- | --- | --- | --- | --- | --- |
| **Atom** | **Atom** | **Length/Å** |  | **Atom** | **Atom** | **Length/Å** |
| S1 | O1 | 1.453(2) |  | C11 | C12 | 1.439(4) |
| S1 | O2 | 1.436(2) |  | C12 | C13 | 1.405(4) |
| S1 | O3 | 1.452(2) |  | C12 | C17 | 1.405(4) |
| S1 | C18 | 1.828(3) |  | C13 | C14 | 1.387(4) |
| F1 | C18 | 1.325(3) |  | C14 | C15 | 1.394(4) |
| F2 | C18 | 1.338(3) |  | C15 | C16 | 1.394(5) |
| F3 | C18 | 1.331(3) |  | C16 | C17 | 1.384(4) |
| S2 | O4 | 1.434(2) |  | N6 | C19 | 1.338(4) |
| S2 | O5 | 1.457(2) |  | N7 | C19 | 1.337(4) |
| S2 | O6 | 1.444(2) |  | N8 | C19 | 1.337(4) |
| S2 | C36 | 1.824(3) |  | N8 | C20 | 1.328(3) |
| F4 | C36 | 1.327(3) |  | N9 | C20 | 1.349(3) |
| F5 | C36 | 1.345(3) |  | N10 | C20 | 1.340(3) |
| F6 | C36 | 1.326(3) |  | N10 | C21 | 1.446(3) |
| N1 | C1 | 1.331(4) |  | C21 | C22 | 1.517(4) |
| N2 | C1 | 1.344(4) |  | C22 | C23 | 1.399(4) |
| N3 | C1 | 1.338(3) |  | C22 | C27 | 1.401(4) |
| N3 | C2 | 1.331(4) |  | C23 | C24 | 1.384(4) |
| N4 | C2 | 1.342(4) |  | C24 | C25 | 1.404(4) |
| N5 | C2 | 1.344(3) |  | C25 | C26 | 1.404(4) |
| N5 | C3 | 1.457(3) |  | C25 | C28 | 1.435(4) |
| C3 | C4 | 1.517(4) |  | C26 | C27 | 1.393(4) |
| C4 | C5 | 1.395(4) |  | C28 | C29 | 1.203(4) |
| C4 | C9 | 1.399(4) |  | C29 | C30 | 1.429(4) |
| C5 | C6 | 1.390(4) |  | C30 | C31 | 1.405(4) |
| C6 | C7 | 1.409(4) |  | C30 | C35 | 1.412(4) |
| C7 | C8 | 1.406(4) |  | C31 | C32 | 1.382(4) |
| C7 | C10 | 1.436(4) |  | C32 | C33 | 1.393(5) |
| C8 | C9 | 1.384(4) |  | C33 | C34 | 1.380(5) |
| C10 | C11 | 1.188(4) |  | C34 | C35 | 1.384(4) |

| **Table S5.** Bond Angles for compound **1b.** | | | | | | | | |
| --- | --- | --- | --- | --- | --- | --- | --- | --- |
| **Atom** | **Atom** | **Atom** | **Angle/˚** |  | **Atom** | **Atom** | **Atom** | **Angle/˚** |
| O1 | S1 | C18 | 103.12(12) |  | C9 | C8 | C7 | 120.4(2) |
| O2 | S1 | O1 | 115.75(14) |  | C8 | C9 | C4 | 120.6(2) |
| O2 | S1 | O3 | 114.93(13) |  | C11 | C10 | C7 | 177.8(3) |
| O2 | S1 | C18 | 103.98(14) |  | C10 | C11 | C12 | 176.5(3) |
| O3 | S1 | O1 | 113.83(13) |  | C13 | C12 | C11 | 119.3(3) |
| O3 | S1 | C18 | 102.92(12) |  | C17 | C12 | C11 | 121.6(3) |
| F1 | C18 | S1 | 111.14(19) |  | C17 | C12 | C13 | 119.2(3) |
| F1 | C18 | F2 | 107.9(2) |  | C14 | C13 | C12 | 120.0(3) |
| F1 | C18 | F3 | 108.8(2) |  | C13 | C14 | C15 | 120.3(3) |
| F2 | C18 | S1 | 110.24(19) |  | C16 | C15 | C14 | 120.0(3) |
| F3 | C18 | S1 | 110.76(19) |  | C17 | C16 | C15 | 120.1(3) |
| F3 | C18 | F2 | 107.8(2) |  | C16 | C17 | C12 | 120.4(3) |
| O4 | S2 | O5 | 114.98(13) |  | C20 | N8 | C19 | 123.0(3) |
| O4 | S2 | O6 | 115.65(13) |  | C20 | N10 | C21 | 124.2(2) |
| O4 | S2 | C36 | 104.31(14) |  | N7 | C19 | N6 | 117.1(3) |
| O5 | S2 | C36 | 102.83(12) |  | N8 | C19 | N6 | 116.8(3) |
| O6 | S2 | O5 | 113.70(13) |  | N8 | C19 | N7 | 126.0(3) |
| O6 | S2 | C36 | 103.12(12) |  | N8 | C20 | N9 | 126.5(3) |
| F4 | C36 | S2 | 111.1(2) |  | N8 | C20 | N10 | 117.7(2) |
| F4 | C36 | F5 | 107.2(2) |  | N10 | C20 | N9 | 115.7(2) |
| F5 | C36 | S2 | 110.04(19) |  | N10 | C21 | C22 | 113.6(2) |
| F6 | C36 | S2 | 111.24(19) |  | C23 | C22 | C21 | 121.5(2) |
| F6 | C36 | F4 | 109.1(2) |  | C23 | C22 | C27 | 118.7(2) |
| F6 | C36 | F5 | 108.1(2) |  | C27 | C22 | C21 | 119.7(2) |
| C2 | N3 | C1 | 123.0(2) |  | C24 | C23 | C22 | 120.8(2) |
| C2 | N5 | C3 | 124.3(2) |  | C23 | C24 | C25 | 120.6(3) |
| N1 | C1 | N2 | 117.7(3) |  | C24 | C25 | C26 | 119.0(2) |
| N1 | C1 | N3 | 116.9(3) |  | C24 | C25 | C28 | 119.9(3) |
| N3 | C1 | N2 | 125.3(3) |  | C26 | C25 | C28 | 121.2(3) |
| N3 | C2 | N4 | 126.7(3) |  | C27 | C26 | C25 | 120.1(2) |
| N3 | C2 | N5 | 117.4(2) |  | C26 | C27 | C22 | 120.8(2) |
| N4 | C2 | N5 | 115.7(3) |  | C29 | C28 | C25 | 177.5(3) |
| N5 | C3 | C4 | 113.6(2) |  | C28 | C29 | C30 | 177.4(3) |
| C5 | C4 | C3 | 121.4(2) |  | C31 | C30 | C29 | 119.9(3) |
| C5 | C4 | C9 | 119.3(2) |  | C31 | C30 | C35 | 118.8(3) |
| C9 | C4 | C3 | 119.3(2) |  | C35 | C30 | C29 | 121.2(3) |
| C6 | C5 | C4 | 120.6(2) |  | C32 | C31 | C30 | 120.2(3) |
| C5 | C6 | C7 | 120.1(3) |  | C31 | C32 | C33 | 120.3(3) |
| C6 | C7 | C10 | 120.0(3) |  | C34 | C33 | C32 | 120.2(3) |
| C8 | C7 | C6 | 118.9(2) |  | C33 | C34 | C35 | 120.4(3) |
| C8 | C7 | C10 | 121.1(3) |  | C34 | C35 | C30 | 120.1(3) |

| **Table S6.** Hydrogen Bonds for compound **1b.** | | | | | | |
| --- | --- | --- | --- | --- | --- | --- |
| **D** | **H** | **A** | **d(D-H)/Å** | **d(H-A)/Å** | **d(D-A)/Å** | **D-H-A/°** |
| N4 | H4A | O4 | 0.78(4) | 2.35(4) | 2.883(3) | 127(3) |
| N4 | H4A | N2 | 0.78(4) | 2.40(4) | 2.875(4) | 121(3) |
| N9 | H9A | O2 | 0.93(4) | 2.17(4) | 2.885(3) | 133(3) |
| N9 | H9A | N7 | 0.93(4) | 2.34(4) | 2.876(4) | 116(3) |
| N4 | H4B | F5^1^ | 0.81(4) | 2.52(4) | 3.112(3) | 131(3) |
| N9 | H9B | F2^2^ | 0.80(5) | 2.48(5) | 3.111(3) | 136(4) |
| N2 | H2A | O3^2^ | 0.95(4) | 2.17(4) | 2.990(3) | 143(3) |
| N7 | H7A | O5^1^ | 0.74(4) | 2.36(4) | 2.997(3) | 146(4) |
| N1 | H1A | O1^3^ | 0.87(4) | 2.07(4) | 2.896(3) | 159(3) |
| N6 | H6A | O6^4^ | 0.82(4) | 2.14(4) | 2.902(3) | 156(3) |
| N2 | H2B | O1^3^ | 0.85(4) | 2.34(4) | 3.075(3) | 145(4) |
| N7 | H7B | O6^4^ | 0.82(4) | 2.37(4) | 3.080(3) | 146(3) |
| N5 | H5 | O3 | 0.81(4) | 2.07(4) | 2.870(3) | 170(4) |
| N10 | H10 | O5 | 0.76(5) | 2.13(5) | 2.868(3) | 165(5) |

^1^+X,-1+Y,+Z; ^2^+X,1+Y,+Z; ^3^-1/2+X,2-Y,+Z; ^4^1/2+X,1-Y,+Z

| **Table S7.** Torsion Angles for compound **1b.** | | | | | | | | | | |
| --- | --- | --- | --- | --- | --- | --- | --- | --- | --- | --- |
| **A** | **B** | **C** | **D** | **Angle/˚** |  | **A** | **B** | **C** | **D** | **Angle/˚** |
| O1 | S1 | C18 | F1 | 59.9(2) |  | C10 | C7 | C8 | C9 | -179.8(2) |
| O1 | S1 | C18 | F2 | 179.48(19) |  | C11 | C12 | C13 | C14 | 179.6(3) |
| O1 | S1 | C18 | F3 | -61.3(2) |  | C11 | C12 | C17 | C16 | -179.6(3) |
| O2 | S1 | C18 | F1 | -61.3(2) |  | C12 | C13 | C14 | C15 | 0.6(4) |
| O2 | S1 | C18 | F2 | 58.3(2) |  | C13 | C12 | C17 | C16 | 0.2(4) |
| O2 | S1 | C18 | F3 | 177.57(18) |  | C13 | C14 | C15 | C16 | -1.1(4) |
| O3 | S1 | C18 | F1 | 178.5(2) |  | C14 | C15 | C16 | C17 | 1.1(5) |
| O3 | S1 | C18 | F2 | -61.9(2) |  | C15 | C16 | C17 | C12 | -0.6(4) |
| O3 | S1 | C18 | F3 | 57.4(2) |  | C17 | C12 | C13 | C14 | -0.2(4) |
| O4 | S2 | C36 | F4 | 60.8(2) |  | N10 | C21 | C22 | C23 | 37.1(3) |
| O4 | S2 | C36 | F5 | -57.8(2) |  | N10 | C21 | C22 | C27 | -145.7(2) |
| O4 | S2 | C36 | F6 | -177.50(18) |  | C19 | N8 | C20 | N9 | -16.0(4) |
| O5 | S2 | C36 | F4 | -178.83(19) |  | C19 | N8 | C20 | N10 | 167.5(2) |
| O5 | S2 | C36 | F5 | 62.6(2) |  | C20 | N8 | C19 | N6 | 158.1(3) |
| O5 | S2 | C36 | F6 | -57.2(2) |  | C20 | N8 | C19 | N7 | -25.1(4) |
| O6 | S2 | C36 | F4 | -60.4(2) |  | C20 | N10 | C21 | C22 | -95.9(3) |
| O6 | S2 | C36 | F5 | -178.96(19) |  | C21 | N10 | C20 | N8 | 0.3(4) |
| O6 | S2 | C36 | F6 | 61.3(2) |  | C21 | N10 | C20 | N9 | -176.5(2) |
| N5 | C3 | C4 | C5 | -37.2(3) |  | C21 | C22 | C23 | C24 | 175.6(2) |
| N5 | C3 | C4 | C9 | 146.0(2) |  | C21 | C22 | C27 | C26 | -175.6(2) |
| C1 | N3 | C2 | N4 | 16.6(4) |  | C22 | C23 | C24 | C25 | 0.3(4) |
| C1 | N3 | C2 | N5 | -167.9(2) |  | C23 | C22 | C27 | C26 | 1.7(4) |
| C2 | N3 | C1 | N1 | -158.3(3) |  | C23 | C24 | C25 | C26 | 1.0(4) |
| C2 | N3 | C1 | N2 | 25.4(4) |  | C23 | C24 | C25 | C28 | -179.0(3) |
| C2 | N5 | C3 | C4 | 96.0(3) |  | C24 | C25 | C26 | C27 | -0.9(4) |
| C3 | N5 | C2 | N3 | -0.2(4) |  | C25 | C26 | C27 | C22 | -0.5(4) |
| C3 | N5 | C2 | N4 | 175.9(2) |  | C27 | C22 | C23 | C24 | -1.6(4) |
| C3 | C4 | C5 | C6 | -176.1(3) |  | C28 | C25 | C26 | C27 | 179.1(3) |
| C3 | C4 | C9 | C8 | 175.6(2) |  | C29 | C30 | C31 | C32 | 180.0(3) |
| C4 | C5 | C6 | C7 | 0.9(4) |  | C29 | C30 | C35 | C34 | 179.9(3) |
| C5 | C4 | C9 | C8 | -1.3(4) |  | C30 | C31 | C32 | C33 | 0.4(4) |
| C5 | C6 | C7 | C8 | -1.8(4) |  | C31 | C30 | C35 | C34 | 0.5(4) |
| C5 | C6 | C7 | C10 | 179.2(3) |  | C31 | C32 | C33 | C34 | -0.1(4) |
| C6 | C7 | C8 | C9 | 1.2(4) |  | C32 | C33 | C34 | C35 | 0.1(4) |
| C7 | C8 | C9 | C4 | 0.3(4) |  | C33 | C34 | C35 | C30 | -0.3(4) |
| C9 | C4 | C5 | C6 | 0.6(4) |  | C35 | C30 | C31 | C32 | -0.5(4) |

| **Table S8.** Hydrogen Atom Coordinates (Å×10^4^) and Isotropic Displacement Parameters (Å^2^×10^3^) for compound **1b.** | | | | |
| --- | --- | --- | --- | --- |
| **Atom** | ***x*** | ***y*** | ***z*** | **U(eq)** |
| H3A | 2763 | 7468 | 3618 | 19 |
| H3B | 3359 | 5241 | 3655 | 19 |
| H5A | 4035 | 10851 | 3892 | 20 |
| H6 | 5179 | 12929 | 3673 | 22 |
| H8 | 5479 | 7971 | 3016 | 21 |
| H9 | 4355 | 5892 | 3243 | 19 |
| H13 | 7144 | 17217 | 3264 | 23 |
| H14 | 8266 | 19594 | 3091 | 27 |
| H15 | 9165 | 18442 | 2680 | 29 |
| H16 | 8973 | 14837 | 2452 | 29 |
| H17 | 7857 | 12444 | 2622 | 25 |
| H21A | 3943 | 9770 | 6328 | 19 |
| H21B | 4539 | 7545 | 6364 | 19 |
| H23 | 3263 | 4166 | 6087 | 20 |
| H24 | 2138 | 2058 | 6307 | 22 |
| H26 | 1801 | 6997 | 6963 | 20 |
| H27 | 2931 | 9106 | 6738 | 19 |
| H31 | 139 | -2288 | 6700 | 22 |
| H32 | -941 | -4722 | 6887 | 27 |
| H33 | -1796 | -3636 | 7313 | 31 |
| H34 | -1568 | -127 | 7553 | 29 |
| H35 | -492 | 2347 | 7372 | 24 |
| H4A | 2330(20) | 9070(60) | 4594(9) | 11(8) |
| H9A | 4970(30) | 5730(70) | 5353(10) | 29(10) |
| H4B | 3040(20) | 7510(60) | 4590(8) | 11(7) |
| H9B | 4330(30) | 7700(80) | 5402(11) | 37(11) |
| H2A | 2600(30) | 12650(70) | 4495(9) | 23(9) |
| H7A | 4820(30) | 2370(70) | 5512(9) | 21(10) |
| H1A | 870(20) | 13750(60) | 4058(8) | 15(8) |
| H6A | 6390(20) | 1250(60) | 5945(8) | 14(8) |
| H2B | 1760(30) | 14040(70) | 4463(9) | 26(10) |
| H7B | 5510(30) | 950(70) | 5535(9) | 20(9) |
| H5 | 3490(20) | 6130(60) | 4160(8) | 16(8) |
| H10 | 3800(30) | 8800(80) | 5836(11) | 39(12) |
| H1B | 970(30) | 11830(80) | 3849(12) | 40(13) |
| H6B | 6340(30) | 3290(80) | 6131(12) | 39(12) |

**Experimental.** Single crystals of C_18_H_18_F_3_N_5_O_3_S were **obtained**. A suitable crystal was selected and **[]** on a **Bruker Venture Metaljet** diffractometer. The crystal was kept at 100 K during data collection. Using Olex, the structure was solved with the XT structure solution program using Intrinsic Phasing and refined with the XL refinement package using Least Squares minimisation.

**Crystal structure determination of 1b**

**Crystal Data** for C_18_H_18_F_3_N_5_O_3_S (*M*=441.43 g/mol): orthorhombic, space group Pca2_1_ (no. 29), *a* = 15.3543(10) Å, *b* = 5.8490(4) Å, *c* = 43.017(3) Å, *V*= 3863.2(4) Å^3^, *Z* = 8, *T* = 100 K, μ(GaKα) = 1.321 mm^-1^, *Dcalc* = 1.518 g/cm^3^, 81637 reflections measured (7.152° ≤ 2Θ ≤ 126.968°), 9587 unique (*R*_int_ = 0.0481, R_sigma_ = 0.0246) which were used in all calculations. The final *R*_1_ was 0.0361 (I > 2σ(I)) and *wR*_2_ was 0.0952 (all data).

**Refinement model description**

Number of restraints - 1, number of constraints - unknown. Details:

1. Twinned data refinement
 Scales: 0.78(2) 0.22(2)
2. Fixed Uiso
 At 1.2 times of:
 All C(H) groups, All C(H,H) groups
3.a Secondary CH2 refined with riding coordinates:
 C3(H3A,H3B), C21(H21A,H21B)
3.b Aromatic/amide H refined with riding coordinates:
 C5(H5A), C6(H6), C8(H8), C9(H9), C13(H13), C14(H14), C15(H15), C16(H16),
 C17(H17), C23(H23), C24(H24), C26(H26), C27(H27), C31(H31), C32(H32), C33(H33),
 C34(H34), C35(H35)

# Measurement of the hydrophobicity of compound 1b

**Experimental**. A 10 µM or 20 µM solution of **1b** was prepared in 5 ml of octanol and mixed with 5 ml distilled water at 25°C The solution was stirred and was left to settle for 24 hours before the absorbance of the octanol fraction was measured. The values obtained were fitted on a calibration curve prepared by measuring the absorbance of **1b** in octanol at various concentrations (2.5, 10, 15, 20 and 30 µM). The logP was calculated using the following formula:

$$logP=\log(\frac{\left[ 1b \right]_{oct}}{\left[ 1b \right]_{water}})$$

Where [**1b**]_oct_ is the concentration of **1b** in octanol and [**1b**]_water_ is the concentration of **1b** in water.

**Table S9**: Calibration curve and Log P values for compound **1b**

| **Concentration of 1b (µM)** | **Absorbance (u.a)** | |  | | |  | |  | |  |  |  |  |  |  |  |
| --- | --- | --- | --- | --- | --- | --- | --- | --- | --- | --- | --- | --- | --- | --- | --- | --- |
| 2.5 | 0.0908 | |  | | |  | |  | |  |  |  |  |  |  |  |
| 10 | 0.3416 | |  | | |  | |  | |  |  |  |  |  |  |  |
| 15 | 0.5334 | |  | | |  | |  | |  |  |  |  |  |  |  |
| 20 | 0.6685 | |  | | |  | |  | |  |  |  |  |  |  |  |
| 30 | 0.9611 | |  | | |  | |  | |  |  |  |  |  |  |  |
|  |  | |  | | |  | |  | |  |  |  |  |  |  |  |
| **Slope** | 0.0317 | |  | | |  | |  | |  |  |  |  |  |  |  |
| **Intercept** | 0.0282 | |  | | |  | |  | |  |  |  |  |  |  |  |
|  |  |  | | |  | | | | |  | | | |  |  |  |
|  | **Absorbance (u.a)** | **[1b] in octanol (µM)** | | **[1b] in water (µM)** | | | **logP** | | | | | | | |  |  |
| Partition 1  (10 µM) | 0.2473 | 6.91 | | 3.09 | | | 0.35 | | 0.42 | | ± | | 0.10 | |  |  |
| Partition 2  (20 µM) | 0.5086 | 15.15 | | 4.85 | | | 0.50 | |  |  |  |  |  |  |  |  |

**
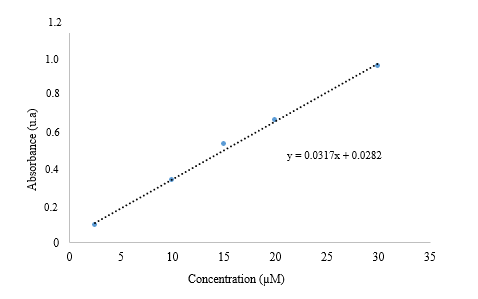
**

**Figure S26**: Calibration curve obtained for the partition of **1b** in octanol

# U-Tube experiments

**Experimental**. U-tube experiment was prepared by adding 800 µL of dichloromethane in a U-shaped tube as a representation of the hydrophobic lipid membrane. On the receiving end (*trans* side) of the tube was added 300 µL of distilled water while the other side (*cis* side) was filled with 300 µL of a 250 µM solution of the compound of interest at 25°C. At 48h and 72h, 100 µL aliquot of the *trans* side was diluted in 900 µL methanol and monitored by LCMS (292 m/z). Area under curve (AUC) was measured and fitted on a calibration curve.

**Table S10**: Calibration curve and U-tube experiment of compound **1b**

| **Concentration of 1b (µM)** | **Average AUC** | | |
| --- | --- | --- | --- |
| **0.125** | 6.11E+05 | ± | 7.71E+04 |
| **1.25** | 3.95E+06 | ± | 4.66E+05 |
| **12.5** | 2.05E+07 | ± | 1.41E+06 |
| **125** | 7.98E+07 | ± | 5.17E+06 |
|  |  |  |  |
| Slope | 6.02E+05 |  |  |
| Intercept | 5.32E+06 |  |  |


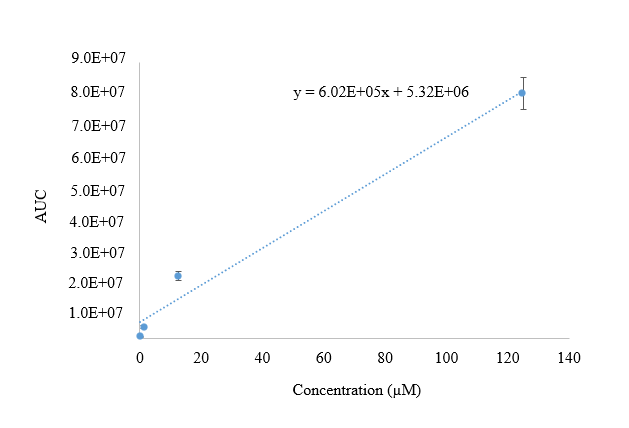


**Figure S27**: Calibration curve obtained for the concentration of **1b** in the *trans* side

**Table S11**: Concentration of compound **1b** in the *trans*-side

|  |  | **AUC** | **Diluted concentration (µM)** | ***Trans*-side concentration (µM)** | | | |
| --- | --- | --- | --- | --- | --- | --- | --- |
| **48h** | **1** | 1,07E+07 | 9,01 | 90,1 | 64,7 | ± | 22,6 |
|  | **2** | 8,76E+06 | 5,71 | 57,1 |  |  |  |
|  | **3** | 8,14E+06 | 4,68 | 46,8 |  |  |  |
| **72h** | **1** | 1,01E+07 | 7,86 | 78,6 | 92,3 | ± | 11,9 |
|  | **2** | 1,13E+07 | 9,93 | 99,3 |  |  |  |
|  | **3** | 1,13E+07 | 9,91 | 99,1 |  |  |  |

# Lucigenin assay

**Experimental**. A 2 mL volume of a 25 mg/mL solution of EYPC in chloroform was slowly reduced *in vacuo* to form a thin film on the side of the flask. Then, 1 mL of a lucigenin solution (2 mM lucigenin, 10 mM Na_2_HPO_4_, 10 mM NaH_2_PO_4_, and 100 mM NaCl) was added, and the resulting suspension was subjected to 10 freeze/thaw cycles (1 cycle = 1 min at -20 °C and 1 min at 37 °C). The mixture was extruded onto a 100-nm polycarbonate membrane 21 times and passed through a Sephadex G-25 column to remove the extravesicular lucigenin. The eluent used for the column was a phosphate buffer with sodium chloride (10 mM Na_2_HPO_4_, 10 mM NaH_2_PO_4_, and 100 mM NaCl), and the resulting liposome solution was diluted to obtain a final concentration of 10 mM. To a quartz cuvette, 2.5 mL phosphate buffer with sodium nitrate (10 mM Na_2_HPO_4_, 10 mM NaH_2_PO_4_, and 100 mM NaNO_3_) and 40 µL of liposome solution were added with light stirring (λ_ex._ = 372 nm, λ_em._ = 503 nm). At *t* = 50 s, a solution of the biguanidium in methanol was added to the cuvette to obtain a 50 mM final solution (50 mol% relative to the concentration of EYPC). At *t* = 300 s, Triton-X 10% v/v was added to lyse the liposomes. The fluorescence was monitored for 350 s.


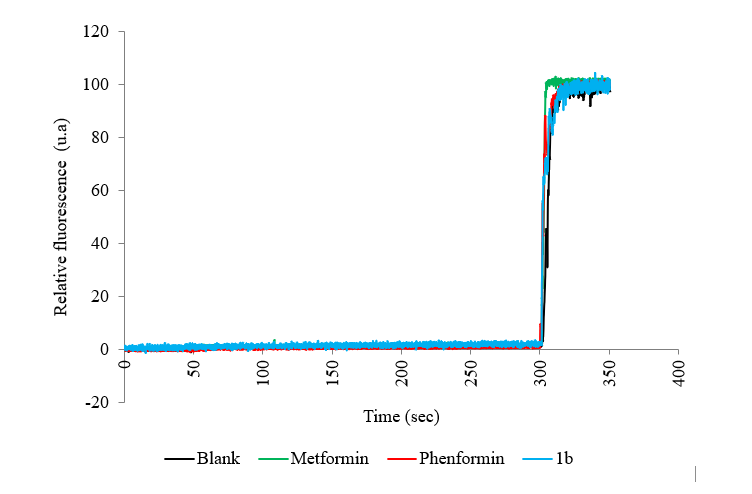


**Figure S28**: Chloride transport in the liposomes

# HPTS assay

**Experimental**. 2 mL of a 25 mg/mL solution of EYPC in chloroform was slowly reduced *in vacuo* to form a thin film on the side of the flask. Then, 1 mL of a solution of the trisodium salt of 8-hydroxypyrene-1,3,6-trisulfonic acid (HPTS) (1 mM HPTS, 10 mM 4-(2-hydroxyethyl)-1-piperazineethanesulfonic acid (HEPES) salt, and 100 mM NaCl, adjusted to pH = 7.4) was added, and the resulting suspension was subjected to 10 freeze/thaw cycles (1 cycle = 1 min at -20 °C and 1 min at 37 °C). The mixture was extruded onto a 100-nm polycarbonate membrane 21 times and passed through a Sephadex G-25 column to remove the extravesicular HPTS. HEPES buffer (10 mM HEPES and 100 mM NaCl, adjusted to pH = 7.4) was used as the eluent for the column and the resulting liposome solution was diluted to obtain a final concentration of 10 mM. To a quartz cuvette, 1.9 mL of HEPES buffer and 25 µL of liposome solution were added with light stirring (λ_ex._ = 405/450 nm, λ_em._ = 510 nm). At *t* = 50 s, a solution biguanidium salt in methanol was added to obtain a 5 mM final concentration. At *t* = 300 s, NaOH was added to obtain a 5 mM final concentration, and, at *t* = 350 s, Triton-X 10% v/v was added to lyse the liposomes. The fluorescence was monitored for 600 s. As a control, we monitored the variation of HPTS fluorescence after the addition of a 100 µg/mL solution of biguanidium salts. No variation was observed.

# Safranin O assay

**Experimental**. 2 mL of a 25 mg/mL solution of EYPC in chloroform was slowly reduced *in vacuo* to form a thin film on the side of the flask. Then, 1 mL of HEPES buffer (10 mM HEPES salt, 100 mM KCl, adjusted to pH = 7.4) was added, and the resulting suspension was subjected to 10 freeze/thaw cycles (1 cycle = 1 min at -20 °C and 1 min at 37 °C). The mixture was extruded on a 100 nm polycarbonate membrane 21 times, and the resulting liposome solution was diluted to obtain a 10 mM final concentration. To a quartz cuvette, 1.9 mL of HEPES buffer with sodium chloride (10 mM HEPES salt and 100 mM NaCl, adjusted to pH = 7.4) and 100 μL of liposome solution were added with light stirring. Safranin O dye was added to a 60 nM final concentration (λ_ex._ = 522 nm, λ_em._ = 581 nm). At *t* = 50 s, a solution of biguanidium salt in DMSO was added to obtain a 100 µg/mL final solution in the cuvette, and the fluorescence was monitored for 300 s. As a control, we monitored the variation in safranin O fluorescence in the solution by adding 100 μg/mL biguanidium salts. No change was observed.

# Mitochondrial permeation and accumulation

**Experimental**. pMXs-3XHA-EGFP-OMP25 retroviral particles were produced in Phoenix cells and then incubated for 8 hours in KP4 cells. Then, positive cells were selected with blasticidine (Santa Cruz Biotechnology, sc-495389). At day 7 post-infection, ∼20 million cells were treated for 3 hours with 15 µM metformin, or 15 µM compound **1b**, or DMSO as vehicle. After treatment, cells were washed twice with PBS and then scraped into 1 mL chilled KPBS (136 mM KCl, 10mM KH_2_PO_4_, pH 7.25) for mitochondrial isolation as described in the litterature^1^, except for the incubation with anti-HA magnetic beads (Thermo Fisher Scientific, 88837), in which the supernatants were incubated with 30 µL of prewashed beads on a vertical-rotating mixer for 1 hour. pMXs-3XHA-EGFP-OMP25 was a gift from David Sabatini (Addgene plasmid # 83356; http://n2t.net/addgene:83356; RRID: Addgene_83356). A standard MTP target plate was used for MALDI-MS analysis (Bruker Daltonics, Billerica, MA). An organic matrix solution of *α*-cyano-4-hydroxycinnamic acid (*α*-CHCA) was best suited for the biguanide drugs and was prepared at a 7 mg/mL concentration in an equal ratio of acetonitrile (ACN) and H_2_O. A 0.5 μL drop of the matrix solution was placed on the target plate for each biological solution analyzed and let to air dry. Then, for each sample, a 0.5 μL drop was pipetted on one of the dried matrix spots and let to air dry. For the MS experiment, an accumulation of 250 shots was obtained for each sample at *m/z* 0-1000 and repeated three times. External calibration was carried out in cubic enhanced mode using known matrix peaks and CsI to obtain five points of calibration over the considered mass range.

1. **Growth assays of pancreatic cell lines**

**Experimental**. KP4 (Riken RCB1005) and PSN1 (ATCC: CRM-CRL-3211), were cultured in Dulbecco's modified Eagle's medium (DMEM) (Wisent, St-Bruno, QC) supplemented with 10 % of fetal bovine serum (FBS) (Wisent). HPNE hTERT (ATCC: CRL1423) were cultured in a mix of 75% DMEM without glucose (Sigma Cat#. D-5030) with additional 2 mM L-glutamine and 1.5 g/L sodium bicarbonate, 25% Medium M3 Base (Incell Corp. Cat# M300F- 500), fetal bovine serum 5% (wisent 10 ng/ml human recombinant EGF (Sigma-Aldrich), 5.5 mM D-glucose (Sigma-Aldrich), 750 ng/ml puromycin (Sigma-Aldrich). For dose-response assays, cells were seeded in 96 well plates (company). After 24 h, treatments were applied and cells were grown for 3 more days. Crystal violet retention assay used for cell growth assays were described previously^2^. The data is expressed as relative absorbance of crystal violet extracted from the cells and diluted in 10 % acetic acid. IC_50_ were determined using Prism (GraphPad). Tumor spheres were grew according to Ferbeyre *et al*^3^.

**Table S12**. IC_50_ of biguanides in KP4 and HPNE cells

|  | |  | **IC_50_** | | | | | |
| --- | --- | --- | --- | --- | --- | --- | --- | --- |
|  | | | KP4 (µM) | | | HPNE (µM) | | |
|  |  | MM (g/mol) |  | | |  | | |
|  | **1a** | 572.50 | 7.55 | ± | 0.07 | 16.12 | ± | 1.73 |
|  | **1b** | 441.43 | 6.13 | ± | 0.13 | 80.91 | ± | 0.44 |
|  | **1c** | 327.82 | 9.21 | ± | 0.71 | 28.09 | ± | 2.85 |
|  | **2a** | 576.53 | 20.72 | ± | 0.81 | 23.21 | ± | 18.16 |
|  | **2b** | 445.43 | 17.42 | ± | 1.18 | 31.26 | ± | 5.65 |
|  | **2c** | 331.85 | 15.83 | ± | 0.80 | 37.44 | ± | 4.25 |
|  | **3a** | 558.51 | 17.00 | ± | 0.19 | 20.18 | ± | 1.24 |
|  | **3b** | 427.43 | 58.27 | ± | 6.14 | 60.41 | ± | 3.20 |
|  | **3c** | 313.82 | 38.91 | ± | 7.49 | 52.61 | ± | 4.87 |
|  | **4a** | 562.51 | 12.21 | ± | 1.08 | 32.62 | ± | 5.65 |
|  | **4b** | 431.43 | 13.90 | ± | 1.57 | 47.39 | ± | 6.21 |
|  | **4c** | 317.82 | 16.87 | ± | 0.48 | 36 ;70 | ± | 3.39 |

# Hemolytic activity

**Experimental**. Red blood cells in Alsever’s solution were centrifuged for 10 min at 300*g*, washed 3 times with PBS buffer, and resuspended in PBS at 2% v/v. To each well of a 96-well plate, 195 µL of red blood cell solution and 5 µL of biguanidium salt in DMSO were added, and the plate was incubated with light agitation for 1 h at 37 °C. The plate was then centrifuged for 10 min at 300*g*, and 50 µL of the supernatant solution of each well was transferred to another plate. Absorbance was measured at λ = 405 nm. Each measurement was performed in triplicate in three different experiments.

**Figure S29** : Hemolytic activity

**Table S13**: Minimal concentration inducing less than 10% hemolysis

|  | **HC 10% (µg/ml)** | **HC 10% (µM)** |
| --- | --- | --- |
| **1a** | > 100 | > 175 |
| **1b** | > 100 | > 227 |
| **1c** | > 100 | > 305 |
| **2a** | > 100 | > 173 |
| **2b** | > 100 | > 225 |
| **2c** | 50 | 151 |
| **3a** | > 100 | > 179 |
| **3b** | > 100 | > 234 |
| **3c** | > 100 | > 319 |
| **4a** | > 100 | > 178 |
| **4b** | > 100 | > 232 |
| **4c** | > 100 | > 315 |

# NAD/NADH quantification

**Experimental**. NAD/NADH quantitation colorimetric kit (#K337-100) from Biovision was used according to manufacturer’s instructions.

1. **Cell death assay**

**Experimental**. Cells were treated for 24h00 with either metformin (5 mM), Phenformin (50 μM), compound 1b (15 μM) or vehicle, in their usual culture medium. At the end of the treatments, adherent cells were recovered by trypsinization, along with floating cells recovered by centrifugation of the culture medium. Floating and adherent cells were pooled together. The faction of dead cells was quantified by counting the proportion of trypan blue-positive (dead cells) and the proportion of living cells (trypan blue-negative).

# Animal experiments

**Experimental.** All experiments were performed in accordance to the rules of the in vivo ethical committe of University of Montreal (CDEA #17-103). 6-7 weeks old female nude mice (Hsd:Athymic Nude- Foxn1nu, Envigo) were implanted sub-cutaneously with 750.000 KP4 cells in 100μl of a sterile mix of 20% Matrigel (Corning) in saline solution. Tumor volume was determined by using a caliper, following the formula 4/3*𝜫*(L*W*T) were L represent the length of the tumor, W, the width, and T, the thickness (all measured in millimeter). For *in vivo* experiments, phenformin was purchased at Sigma-Aldrich (P7045). The compounds were dissolved in the vehicle: 45% Propyleneglycol (Sigma-Aldrich) +5% Tween 80 (Sigma-Aldrich) + ddH2O. Mice were injected daily intraperitoneally (in 100 μl), 5 days/week starting day 11 post engraftment with either phenformin at 50 mg/kg/d, compound **1b** at 50 mg/kg/d or with vehicle.

# Immunoblots

**Experimental.** Cells were rinsed twice with 1X PBS, and lysed in Laemmli 2x (4% SDS, 20% glycerol, 120 mM Tris-HCl pH 6.8). Laemmli extracts were boiled for 5 minutes, then the concentration was assessed with NanoDrop (ThermoFisher) and equalized between samples. Lysates were supplemented with 10% beta-mercaptoethanol and 0.05% bromophenol blue and boiled again for 5 minutes. 25 µg of extracts were loaded on SDS-PAGE then transferred on nitrocellulose membranes for 1h30 at 130V. Membranes were rinsed in 1XPBS1-0.05% Tween-20 then blocked for 1h in 1XPBS, 0.05%-Tween-20 5% Milk. Membranes were rinsed again 3 x 10 min in PBS-Tween, then incubated overnight at 4°C with the following primary antibodies in 1XPBS-0.1%BSA, 0.02%Sodium azide: anti-phospho-AMPK T172 (1:1000, Rabbit, 2531S, Cell Signaling), anti-AMPKalpha (1:1000, Rabbit, 2532, Cell Signaling), anti-phospho-ACC S79 (1:1000, Rabbit Polyclonal, 3661S, Cell Signaling), or incubated for 1h at RT with anti-beta-Actin (1:2000, Mouse, 3700, Cell Signaling). Membranes were washed 3 x 10 min in PBS-Tween, then incubated with the corresponding secondary antibody diluted in PBS-Tween-1%Milk for 1h at RT: anti-mouse IgG conjugated to HRP (1:3000, Goat, 170-6516, BioRad), anti-rabbit conjugated to HRP (1:3000, Goat, 170-6515, BioRad). Membranes were washed 3 x 10 min with PBS-Tween then revealed with ECL reagent (GE Healthcare Life Sciences, #RPN2106) by exposition with autoradiographic films.

1. **Immunofluorescence**

**Experimental**. KP-4 cells were seeded at 50% confluency on slides in 6-well plates. The day after, the media was changed for media containing either Metformin hydrochloride 5mM, Compound 1b 15µM or DMSO as vehicle. After 24h of treatment, cells were washed twice with PBS1X then were fixed during 10 minutes at 4°C with 4% paraformaldehyde. Cells were washed again three times with PBS1X then permeabilized during 5 min in PBS1X, 2%BSA, 0,2%Triton X-100. Slides were washed three times 10 minutes with 1XPBS, 2%BSA, then incubated overnight with primary antibody anti-TOMM20 (Rabbit, 1:150, Santa Cruz, sc-11415) diluted in 1XPBS, 2%BSA. Slides were washed three times 10 minutes with 1XPBS, 2%BSA then incubated 1 hour at RT with anti-Rabbit-AlexaFluor568 secondary antibody (Goat, 1:1000, ThermoFisher Scientific). Cells were washed four times 10 minutes with 1XPBS then mounted with Vectashield mouting media containing DAPI (Vector laboratories H-1200). Imaging of the slides was done on epifluorescence microscope Zeiss Axio-Imager Z2.

1. **Seahorse experiments**

**Experimental**. ECAR (extracellular acidification rate) and OCR of KP4 cells (oxygen consumption rate) were measured on a Seahorse XF-24 (Agilent). The day before the experiment, KP4 cells were seeded in XF24 FluxPak seahorse plates (Agilent) at a density of 40.000 cells per well, in usual KP4 culture medium (DMEM, 10% FSB). For the experiment, the culture medium of KP4 cells was replaced by 600 μl of Seahorse medium per well: DMEM (Wisent #219-060-XK) supplemented with 11 mM of D-Glucose, 2mM of L-Glutamine and 5% of FBS. OCR and ECAR were followed for 180 minutes (140 minutes after injection of the different compounds).

**References**

1 Chen, W. W., Freinkman, E., Wang, T., Birsoy, K. & Sabatini, D. M. Absolute Quantification of Matrix Metabolites Reveals the Dynamics of Mitochondrial Metabolism. *Cell* **166**, 1324-1337 e1311, doi:10.1016/j.cell.2016.07.040 (2016).

2 Lessard, F. *et al.* Senescence-associated ribosome biogenesis defects contributes to cell cycle arrest through the Rb pathway. *Nat Cell Biol* **20**, 789-799, doi:10.1038/s41556-018-0127-y (2018).

3 Deschenes-Simard, X. *et al.* Circumventing senescence is associated with stem cell properties and metformin sensitivity. *Aging Cell*, e12889, doi:10.1111/acel.12889 (2019).
